# Comparative efficacy of different scoliosis-specific exercise protocols on cobb angle in adolescent idiopathic scoliosis: a network meta-analysis

**DOI:** 10.3389/fspor.2026.1847841

**Published:** 2026-06-25

**Authors:** Penglong Wang, Yongping Xi, Xiaozan Wang

**Affiliations:** 1 Graduate School, Shenyang Sport University, Liaoning, China; 2 College of Physical Education, Zhangjiakou University, Hebei, China; 3 College of Physical Education and Health, East China Normal University, Shanghai, China

**Keywords:** adolescent idiopathic scoliosis, exercise protocol, exercise type, frequency, network meta-analysis

## Abstract

**Objective:**

To compare the efficacy of different scoliosis-specific exercise protocols on the Cobb angle in adolescent idiopathic scoliosis (AIS) using a network meta-analysis, and to rank the five key prescription parameters—exercise type, intervention period, session duration, frequency, and intensity—within a unified evidence framework, thereby moving beyond the traditional question of “whether exercise works” to address “which parameter combinations have the highest probability of producing optimal improvement.”

**Methods:**

A systematic search was performed in PubMed, Web of Science, Embase, Cochrane Library, CNKI, Wanfang Data, and VIP databases for randomized controlled trials from inception to December 2025. Two researchers independently screened studies, extracted data, and assessed risk of bias. RevMan 5.3 and Stata 16.0 were used for data analysis.

**Results:**

A total of 24 studies involving 1,149 patients were included. The network meta-analysis showed that: regarding exercise type, Schroth combined with sensory integration training showed the greatest effect size [MD = −8.01, 95% CI (−11.47, −4.54)]; regarding intervention period, 12–18 weeks showed the greatest effect size [MD = −3.67, 95% CI (−5.52, −1.81)]; regarding session duration, 60 min showed the greatest effect size [MD = −4.00, 95% CI (−5.76, −2.23)]; regarding frequency, three times per week showed the greatest effect size [MD = −3.55, 95% CI (−4.76, −2.35)]; regarding intensity, moderate intensity showed the greatest effect size [MD = −3.78, 95% CI (−5.99, −1.57)]. The SUCRA rankings placed these parameters first in their respective networks.

**Conclusion:**

Based on probability ranking, Schroth combined with sensory integration training, delivered at moderate intensity, three times per week, 30–60 min per session, for 12–18 weeks, is likely to be among the most effective protocols for improving the Cobb angle in AIS. However, this finding is derived from a limited number of studies, and each protocol component is supported by sparse evidence within its respective network. These results should be interpreted with caution and must be individualized in clinical practice.

**Systematic Review Registration:**

identifier PROSPERO CRD420251249777.

## 1 Introduction

Adolescent idiopathic scoliosis (AIS) is a three-dimensional structural deformity of the spine that occurs during growth and development. It is diagnosed by a coronal Cobb angle >10° and is often accompanied by vertebral rotation and abnormal sagittal curvature (1, 2). AIS has emerged as a significant health concern affecting the physical and mental well-being of adolescents, with a global prevalence ranging from approximately 0.47% to 5.2% (3, 4). The condition not only leads to cosmetic deformities such as shoulder asymmetry, trunk imbalance, and pelvic tilt; in severe cases, particularly in patients with large thoracic curves, it may also compromise cardiopulmonary function, and can lead to back pain and psychological distress including anxiety and low self-esteem (5), and may adversely affect social functioning and psychological well-being in some affected adolescents during this critical developmental period. Because the pathogenesis of AIS remains unclear and may involve multiple factors such as genetics, biomechanics, hormonal levels, and neurological abnormalities, exploring safe and effective non-surgical interventions is of great clinical and social importance for controlling curve progression and improving patients' quality of life.

According to the current guidelines of the International Society on Scoliosis Orthopaedic and Rehabilitation Treatment (SOSORT), conservative treatment is recommended for AIS patients with a Cobb angle <45° (6). Specifically, for mild curves (Cobb angle 10°–20°), scoliosis-specific exercise therapy with regular follow-up observation is typically recommended. When the Cobb angle exceeds 20°–25° and the patient remains skeletally immature, brace treatment should be added to exercise therapy to maximize the control of curve progression. Among these, exercise therapy has become a research hotspot in conservative treatment for AIS in recent years because of its advantages of active participation, few side effects, and potential to improve postural control and quality of life. Numerous studies have confirmed the efficacy of various specific exercise therapies for AIS, such as the Schroth method (originating in Germany), the Lyon method (France), the Scientific Exercise Approach to Scoliosis (SEAS, Italy), and the Barcelona Scoliosis Physical Therapy School (BSPTS) approach (3, 7, 8). Based on different theoretical frameworks and training principles, these therapies aim to improve spinal symmetry and function through three—dimensional self—correction, breathing exercises, core muscle activation, and postural control. In addition to single—modality therapies, an increasing number of studies have begun to explore combined treatment protocols, such as Schroth exercises combined with hippotherapy, suspension training, or balance training, in order to achieve better clinical outcomes (7, 9).

However, the existing evidence base has three key structural gaps. First, previous studies have predominantly focused on whether exercise therapy works, confirming efficacy through pairwise comparisons (3, 10, 11), but have rarely addressed which combination of prescription parameters is most likely to be optimal. Clinicians thus know that exercise is effective, yet lack evidence-based guidance on how to combine exercise type, period, session duration, frequency, and intensity for maximal benefit. Second, previous meta-analyses have generally treated exercise interventions as holistic packages rather than decomposing them into independent prescription variables. This bundled approach directly limits the precision of dosage interpretation: it remains unclear whether the treatment effect is driven by the exercise type itself or by a specific duration or frequency. Third, as a consequence of these structural deficiencies, current clinical practice relies heavily on empirical judgment. Faced with more than 17 exercise types and multiple dosage combinations, rehabilitation therapists lack a unified, probability-based reference framework to guide individualized prescription. Based on these gaps, the present study employs a network meta-analysis that systematically decomposes exercise protocols into five independent dimensions—type, period, session duration, frequency, and intensity—for simultaneous comparison and probability ranking within a unified evidence framework. This design moves beyond the question of “whether exercise works” to address “which parameter combinations have the highest probability of producing optimal Cobb angle improvement,” thereby building a bridge from empirical decision-making toward evidence-based clinical practice.

## 2 Materials and methods

### 2.1 Protocol registration

This study was conducted in accordance with the Preferred Reporting Items for Systematic Reviews and Meta-Analyses (PRISMA) statement (12) and was registered on the Prospective Register of Systematic Reviews (PROSPERO) (CRD420251249777).

### 2.2 Search strategy

A computerized search was performed in the following databases: PubMed, Web of Science, Embase, China National Knowledge Infrastructure (CNKI), Wanfang Data, and VIP (China Science and Technology Journal Database). The search period covered from the inception of each database to December 21, 2025. A combination of MeSH terms and free words from the PubMed MeSH vocabulary was used, and the search strategy was adapted to the characteristics of each database. English search terms included Scoliosis, Exercise, Schroth Exercises, Lyon Exercise, Pilates Exercises, Barcelona Scoliosis Physical Therapy School, Physiotherapy scoliosis-specific exercise, Schroth and Sensory Integration Training, Schroth Exercise and Hippotherapy Training, and Randomized controlled trial. The detailed search strategy for PubMed is presented in Table 1.

**Table 1:** Search strategy.

Database	Search Strategy
PubMed	(((“Scoliosis”[Mesh]) OR (Adolescent Idiopathic Scoliosis[Title/Abstract])) AND ((“Exercise”[Mesh]) OR (((((((((((((((((((((((((Exercises[Title/Abstract]) OR (Exercise, Physical[Title/Abstract])) OR (Schroth Exercise[Title/Abstract])) OR (Lyon Exercise[Title/Abstract])) OR (Pilates Exercises[Title/Abstract])) OR (Scientific Exercise，Approach to Scoliosis[Title/Abstract])) OR (Core Stabilization Exercise[Title/Abstract])) OR (Barcelona Scoliosis Physical Therapy School[Title/Abstract])) OR (Physiotherapy Scoliosisspecific Exercise[Title/Abstract])) OR (Functional Individual Therapy of Scoliosis[Title/Abstract])) OR (Yoga[Title/Abstract])) OR (Proprioceptive Neuromuscular Facilitation[Title/Abstract])) OR (Combination Therapy[Title/Abstract])) OR (Schroth Exercise and Hippotherapy Training[Title/Abstract])) OR (Physiotherapy Scoliosis-Specific Exercise[Title/Abstract])) OR (Schroth Exercise and Sling Training[Title/Abstract])) OR (Schroth Exercise and Balance Training[Title/Abstract])) OR (Pelvic Rotation Correction and Schroth Exercise[Title/Abstract])) OR (Daoyin Spinal Balance Exercises[Title/Abstract])) OR (Active Self-correction and Task-oriented Exercises[Title/Abstract])) OR (Side-alternating Whole Body Vibration[Title/Abstract])) OR (Schroth and Sensory Integration Training[Title/Abstract])) OR (Spinal Strengthening Exercises[Title/Abstract])) OR (Activity, Physical[Title/Abstract])) OR (Physical Activities[Title/Abstract])))) AND (Randomized controlled trial[publication type] or randomized[title/abstract] or placebo[title/abstract])

### 2.3 Literature inclusion and exclusion criteria

The inclusion and exclusion criteria were established based on the PICOS framework (13). The inclusion criteria were as follows: (1) Study design: randomized controlled trial. (2) Participants: adolescents diagnosed with AIS, aged 8 to 18 years, with a Cobb angle ranging from 10° to 45° (mild to moderate). Risser sign was not restricted.(3) Intervention and comparator: The experimental group received an exercise protocol—based therapy; the control group received either a different exercise protocol—based therapy or routine care (4). Outcomes: The primary outcome measure was the Cobb angle. Data on exercise protocol variables—including type of exercise, intervention period, session duration, frequency, and intensity—were extracted.

The exclusion criteria included: (1) Non—randomized studies (e.g., cohort studies, case—control studies, quasi—experimental designs, case reports, and case series) were excluded because they are subject to high selection bias and confounding, which would compromise the validity of causal inference in a network meta—analysis. (2) Animal experiments, as their findings cannot be directly extrapolated to human AIS patients. (3) Systematic reviews, meta—analyses, conference abstracts, editorials, commentaries, and letters were excluded because they do not provide original primary data, and conference abstracts often lack detailed methodological information and outcome statistics (e.g., means, standard deviations). (4) Studies with incomplete or insufficient outcome data (e.g., missing means, standard deviations, sample sizes, or Cobb angle change values) that could not be obtained from the authors were excluded. (5) Duplicate publications reporting on the same study cohort were excluded; only the study with the largest sample size or most complete data was retained. (6) Studies in which participants received concurrent bracing or other non—exercise conservative treatments (e.g., electrical stimulation, manual therapy) during the exercise intervention were excluded, because such co—interventions would confound the isolated effect of the exercise protocol. (7) Studies that did not report the Cobb angle as an outcome measure or reported only long—term follow—up data without a post—intervention assessment immediately after the exercise period were excluded. (8) Studies involving participants with previous spinal surgery were excluded.

### 2.4 Study selection and data extraction

The NoteExpress reference management software was used to manage the retrieved documents. Two researchers (who had received independent training) independently performed literature screening and data extraction in a back—to—back manner. In the event of disagreement, a third researcher was consulted to reach a consensus through discussion. After data extraction was completed, cross—verification was carried out. The extracted data included the basic information of the included studies, exercise protocol variables (type, period, session duration, frequency, intensity), and outcome measures. Since the included studies did not directly report exercise intensity, the intensity was graded in this study based on indirect indicators: (1) range of repetitions; (2) hold time; (3) whether progressive phases were used; (4) whether resistance/loading was used; (5) session duration and frequency. The grading criteria were as follows: low intensity—no progressive load and ≤10 repetitions, or the study explicitly described the intervention as low intensity; moderate intensity—progressive load with 10–30 repetitions or hold time <10 s; moderate—to—high intensity—≥30 repetitions or hold time ≥10 s, or explicit use of resistance/loading. It should be noted that none of the included studies directly reported objective physiological indicators of exercise intensity (e.g., percentage of heart rate reserve, percentage of one-repetition maximum, or ratings of perceived exertion). Therefore, the intensity classification in the present study was derived indirectly from exercise prescription dose parameters and represents a proxy categorization, which may not be fully equivalent to directly measured exercise intensity. This classification method has inherent limitations that should be considered when interpreting the results.

### 2.5 Risk of bias evaluation of included studies

The risk of bias of the included studies was assessed using RevMan 5.3 software according to the Cochrane risk of bias tool for randomized controlled trials.

### 2.6 Statistical methods

Statistical analyses were performed using Stata 16.0 software. Network evidence plots were generated for each component of the different exercise protocols. The results of the network meta—analysis were presented using league tables for pairwise comparisons. Cumulative probability ranking plots were drawn for exercise type, period, session duration, frequency, and intensity to identify which protocols had the highest probability of being most effective for evidence—based treatment of adolescent idiopathic scoliosis. The mean difference (MD) was used as the effect measure, with its 95% confidence interval (CI). Inconsistency was assessed using the node—splitting method. If the difference between direct and indirect comparisons was not statistically significant (*P* > 0.05), a consistency model was used; otherwise, an inconsistency model was applied. Publication bias or small—study effects were examined using an adjusted comparison funnel plot and Egger's test. Given that the statistical power of the Egger test may be limited in the context of network meta-analysis, the symmetry of the comparison-adjusted funnel plot was also qualitatively assessed to provide a more comprehensive evaluation of publication bias risk. In addition, the *Q*-test and *I*^2^ statistic were used to assess between-study heterogeneity, with I^2^ > 50% indicating substantial heterogeneity. To explore potential sources of heterogeneity, subgroup analyses were performed according to the overall category of exercise intervention. In this study, in the analyses of the three dimensions—exercise period, frequency, and session duration—the control groups across different studies were defined as the same category. As a result, the corresponding evidence networks were star-shaped or tree-like structures, for which formal inconsistency testing could not be performed. For these networks without closed loops, the proportion of indirect evidence is relatively high. Therefore, the obtained SUCRA rankings should be regarded as highly exploratory and interpreted with substantial caution.

## 3 Results

### 3.1 Results of literature search

**Figure 1:**
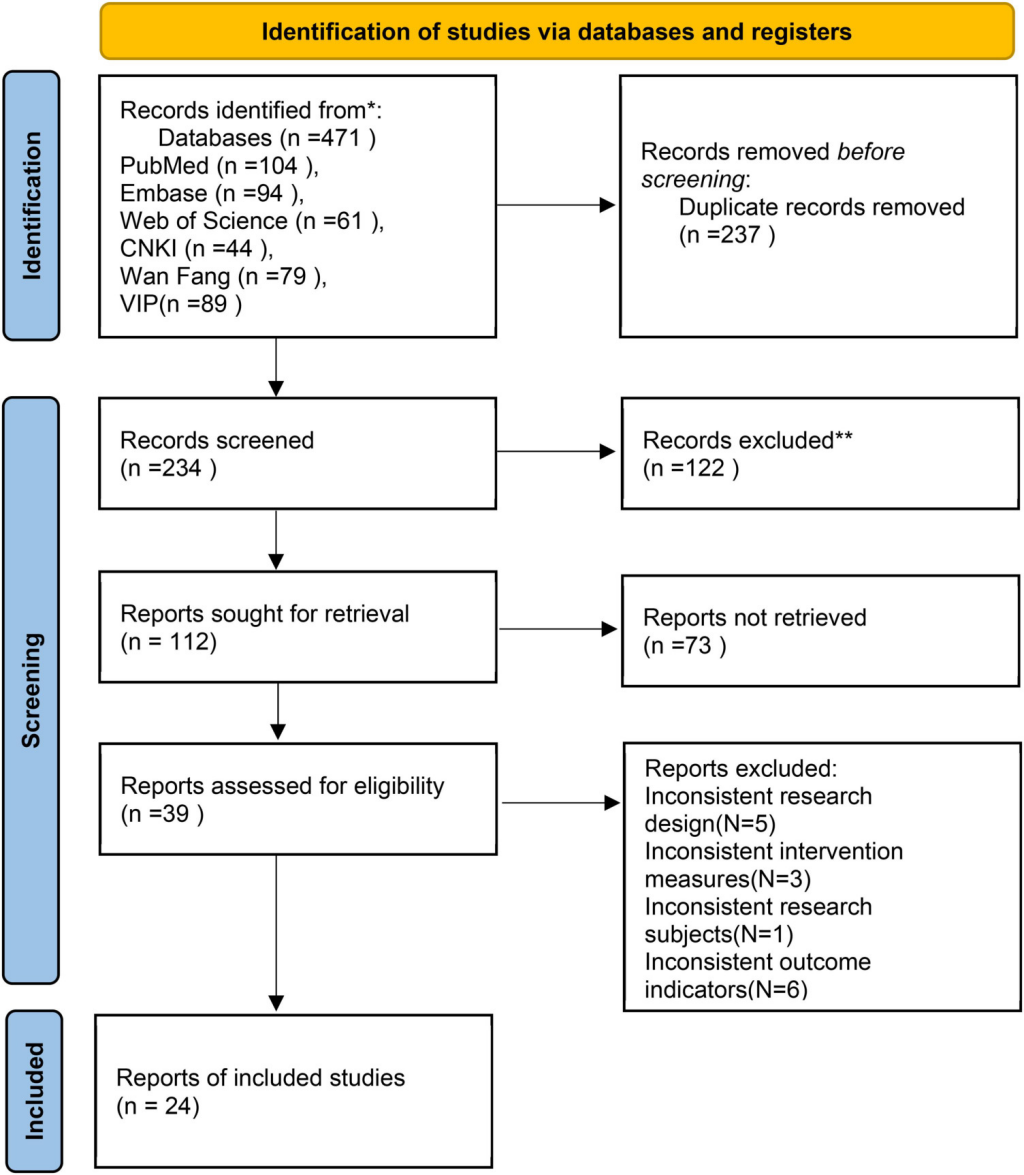
Flowchart of literature selection.

A total of 471 records were identified through the initial search. After stepwise screening, including initial screening and full-text reading, 24 RCTs involving 1,149 patients were finally included. Figure 1 presents the literature screening process and results.

### 3.2 Basic characteristics of the included literature

A total of 24 studies (14–37) were included in this study, with a total sample size of 1,149 patients (592 in the experimental group and 557 in the control group). Among the included studies, six were from China, five from Turkey, three from South Korea, two from the United States, and one each from Egypt, Germany, Canada, Serbia, Saudi Arabia, Greece, Italy, and India. The basic characteristics of the included studies are presented in Table 2.

**Table 2:** Characteristics of the included studies.

Study	Country	Year	Sample		Mean Age		Exercise		S	F	P	I	Outcomes
			T	C	T	C	T	C					
Gözde Gür (14)	Turkey	2017	12	13	14.20 ± 1.80	14.00 ± 1.60	CS	TR	60	2	10	M	①
Sang-Hee Won (15)	Korea	2021	10	10	14.50 ± 2.50	15.90 ± 2.69	PNF	TR	30	3	26	L	①
R.A. Mohamed (16)	Egypt	2021	17	17	14.50 ± 1.20	14.90 ± 1.40	SC	PNF	60	3	26	M	①
Karina Amani Zapata (17)	USA	2023	35	22	11.60 ± 1.10	12.50 ± 1.40	SC	Con	15	5	52	L	①
Abdel-aziem (18)	Saudi Arabia	2022	27	25	14.74 ± 1.79	15.04 ± 1.81	SH	SC	60	3	10	M-H	①
Oznur Büyükturan (19)	Turkiye	2024	15	16	14.00 ± 1.90	14.20 ± 2.00	SC	LY	90	3	26	M	①
Mehmet Hanifi Kaya (20)	Turkiye	2025	34	33	13.80 ± 1.60	14.10 ± 1.80	SC	PNF	60	3	26	M	①
Xiangyu Shen (21)	China	2023	30	29	13.47 ± 1.01	13.75 ± 1.02	SB	SC	90	3	6	M	①
Wei Hui (22)	China	2015	58	49	9.10 ± 0.40	8.90 ± 0.60	DS	Con	80	7	52	L	①
Peng Zhang (23)	China	2024	31	29	13.42 ± 1.06	13.97 ± 1.21	SS	SC	90	3	12	M	①
Vanja Dimirijevi'c (24)	Serbia	2025	17	17	14.11 ± 1.02	13.41 ± 1.63	SC	TR	90	3	8	M	①
Arvind kumar (25)	India	2017	18	18	12.17 ± 1.72	11.56 ± 1.46	AS	SSE	50	7	52	M	①
S. Langsiepen (26)	Germany	2017	20	18	13.60 ± 1.60	14.00 ± 0.90	SWBV	TR	12	5	26	M	①
Gichul Kim (27)	Korea	2016	12	12	15.60 ± 1.10	15.30 ± 0.80	SC	PE	60	3	12	M	①
Athanasios Kyrkousis (28)	Greece	2024	38	39	13.67 ± 1.19	13.55 ± 1.02	SC	Con	60	3	12	M	①
Marco Monticone (29)	Italy	2014	55	55	12.50 ± 1.10	12.40 ± 1.10	AS	TR	60	3	12	M	①
Sanja Schreiber (30)	Canada	2016	21	23	13.30 ± 0.60	13.50 ± 0.80	SC	Con	45	7	26	M	①
Hikmet Kocaman (31)	Turkey	2021	14	14	14.07 ± 2.37	14.21 ± 2.19	SC	CS	90	3	10	M	①
Yafei Zhang (32)	China	2024	21	21	13.10 ± 2.23	13.57 ± 2.60	SR	SC	90	1	24	M	①
Sang-Gil Lee (33)	Korea	2025	14	14	11.57 ± 1.70	12.07 ± 1.00	SC	Con	50	3	12	M	①
Wei Liang (34)	China	2025	30	30	13.00 ± 0.32	13.47 ± 0.26	SSIT	SC	90	3	12	M	①
Gozde Yagci (36)	Turkey	2018	15	15	14.00 ± 1.30	14.20 ± 1.50	CS	SEAS	20	7	18	M	①
Karina A. Zapata (35)	USA	2019	19	14	12.50 ± 1.50	11.80 ± 0.90	BSPT	Con	15	3	52	M	①
Yu Zheng (37)	China	2018	29	24	12.40 ± 0.90	12.30 ± 0.80	SEAS	Con	15	7	52	M-H	①

CS, core stabilization exercises; TR, traditional rehabilitation exercises; PNF, proprioceptive neuromuscular facilitation; SC, Schroth; SH, schroth & hippotherapy exercises; LY, Lyon; SB, schroth & balance training; DS, daoyin spinal balance exercises; SS, schroth & sling; SSE, spinal strengthening exercises; SWBV, side-alternating whole body vibration; PE, pilates exercise; AS, active self-correction and task-oriented exercises; SR, schroth & rehabilitation exercises; SSIT, schroth & sensory integration training; SEAS, scientific exercise approach to scoliosis; BSPT, barcelona scoliosis physical therapy school; Con, standard of care. T, Intervention group; C, Control group; S, Session; F, Frequency; P, Period; I, Intensity; ①, Cobb Angle.

### 3.3 Evaluation of the quality of the included literature

**Figure 2:**
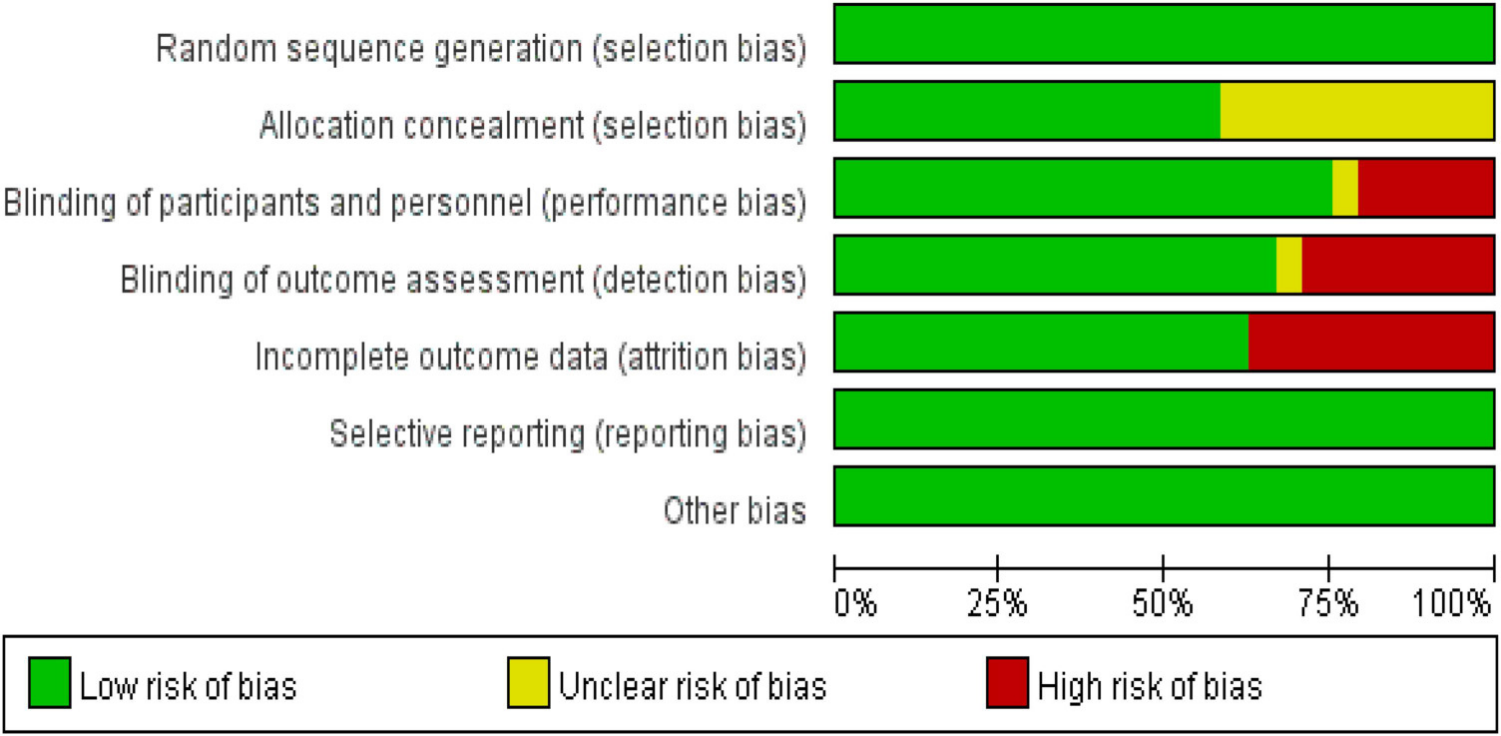
Overall bias risk diagram.

**Figure 3:**
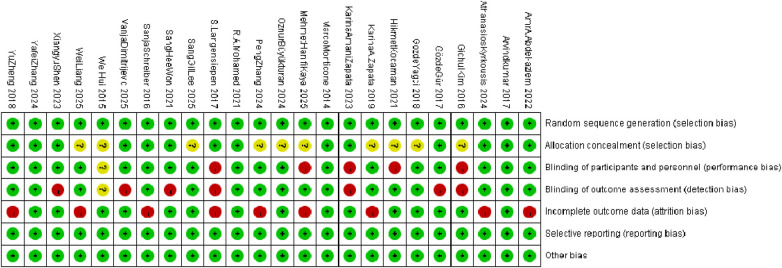
Bias risk diagram for each item.

The methodological quality of the included studies was rigorously assessed using the Cochrane Risk of Bias tool, implemented in RevMan 5.4 software (38). The detailed risk of bias judgments for each study are presented in Figure 2, and a summary of the overall distribution of these risks across all studies is provided in Figure 3. For the specific domain of “random sequence generation”, studies were rated as “low risk”if an appropriate randomization method (e.g., using a random number table) was explicitly described. Studies that only mentioned being “randomized” without detailing the method were judged as “unclear risk”. Those employing a non-random allocation method were categorized as “high risk”.

### 3.4 Results of network meta-analysis

#### 3.4.1 Network evidence diagram

**Figure 4:**
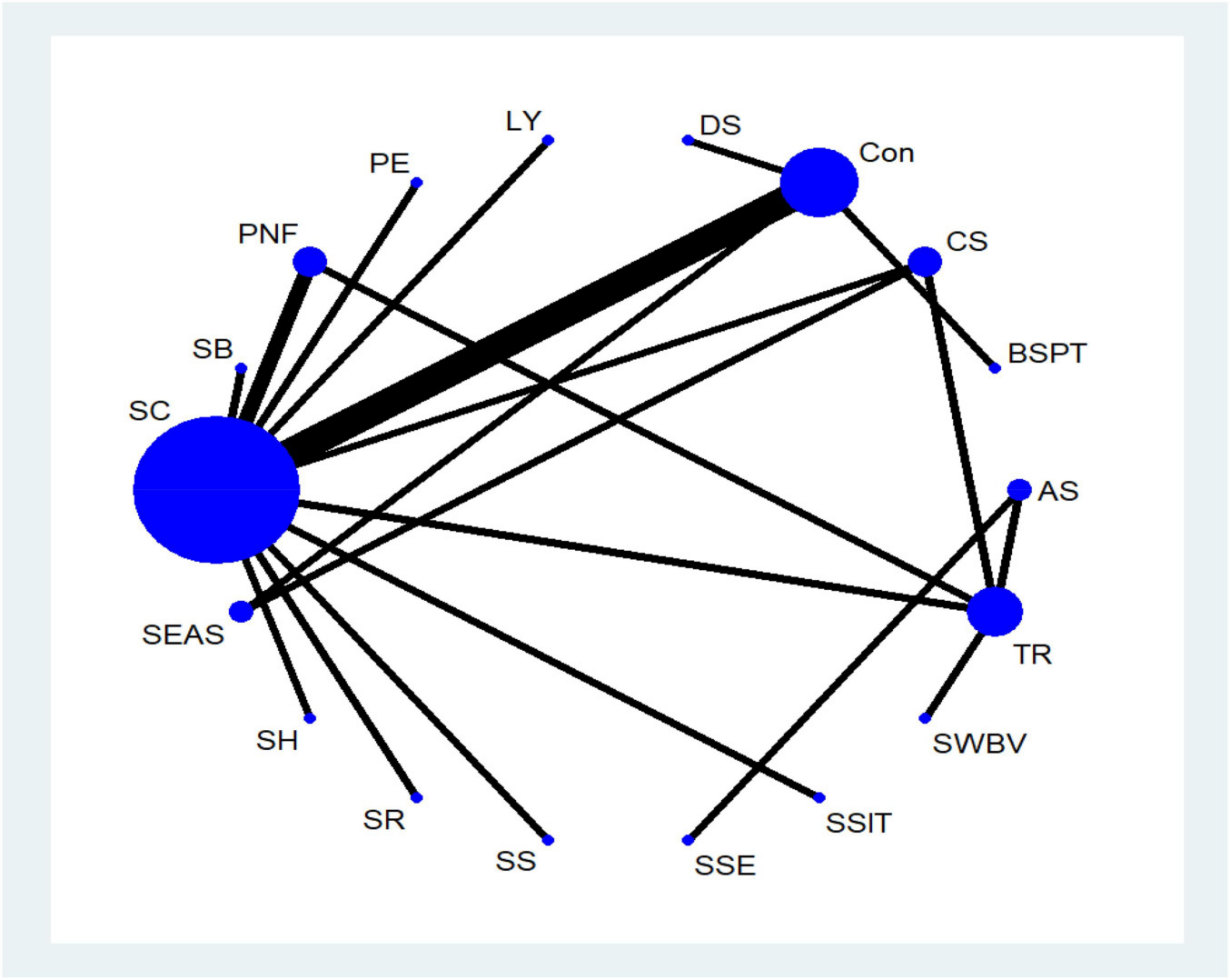
Exercise type network evidence diagram. CS, core stabilization exercises; TR, traditional rehabilitation exercises; PNF, proprioceptive neuromuscular facilitation; SC, Schroth; SH, schroth & hippotherapy exercises; LY, Lyon; SB, schroth & balance training; DS, daoyin spinal balance exercises; SS, schroth & sling; SSE, spinal strengthening exercises; SWBV, side-alternating whole body vibration; PE, pilates exercise; AS, active self-correction and task-oriented exercises; SR, schroth & rehabilitation exercises; SSIT, schroth & sensory integration training; SEAS, scientific exercise approach to scoliosis; BSPT, Barcelona scoliosis physical therapy school; Con, standard of care.

**Figure 5:**
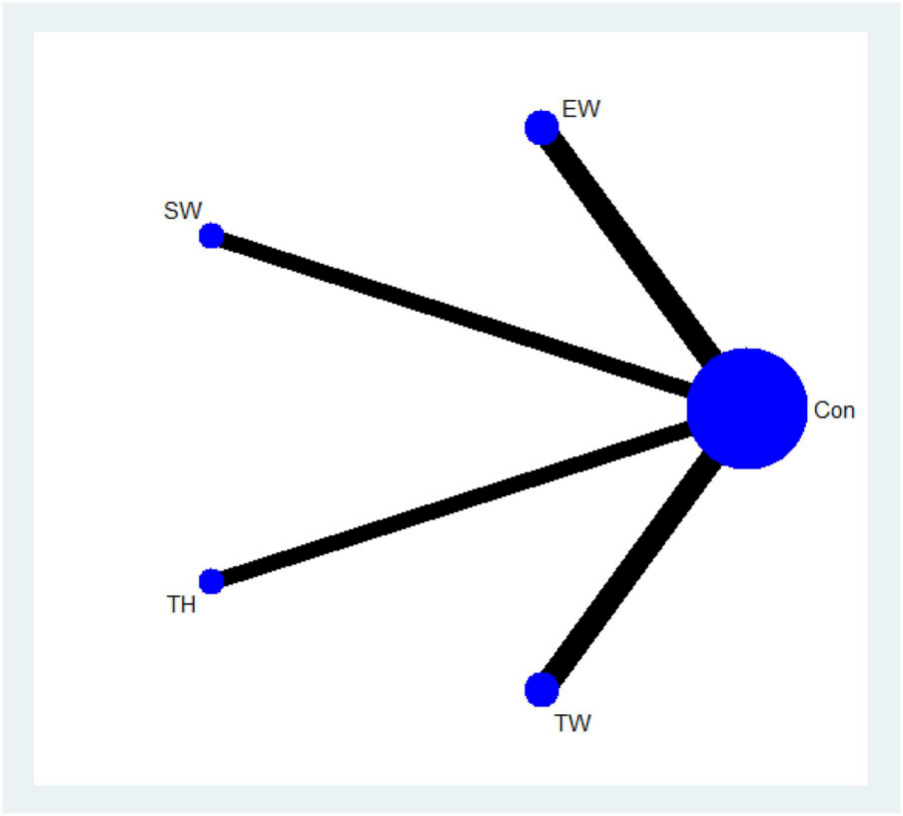
Intervention period network evidence diagram. SW, 6–10 Weeks; EW, 12–18 Weeks; TW, 24–26 Weeks; TH, 52 Weeks; Con, Intervention group.

**Figure 6:**
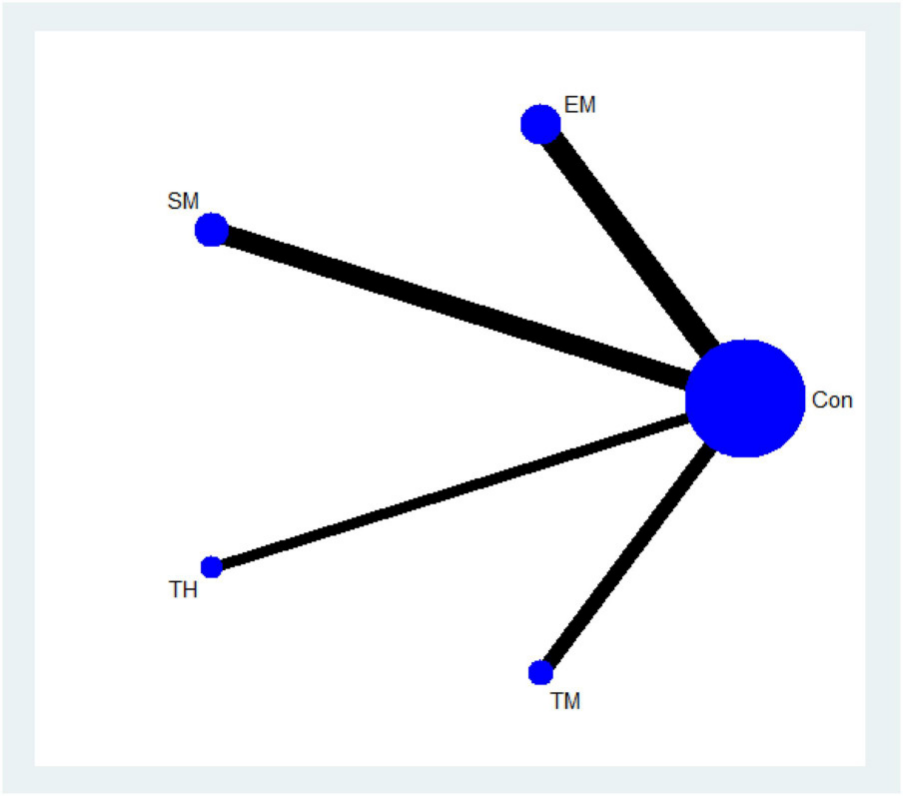
Session duration network evidence diagram. TM, 12–20 Min; TH, 30–45 Min; SM, 60 Min; EM, 80–90 Min; Con, Intervention group.

**Figure 7:**
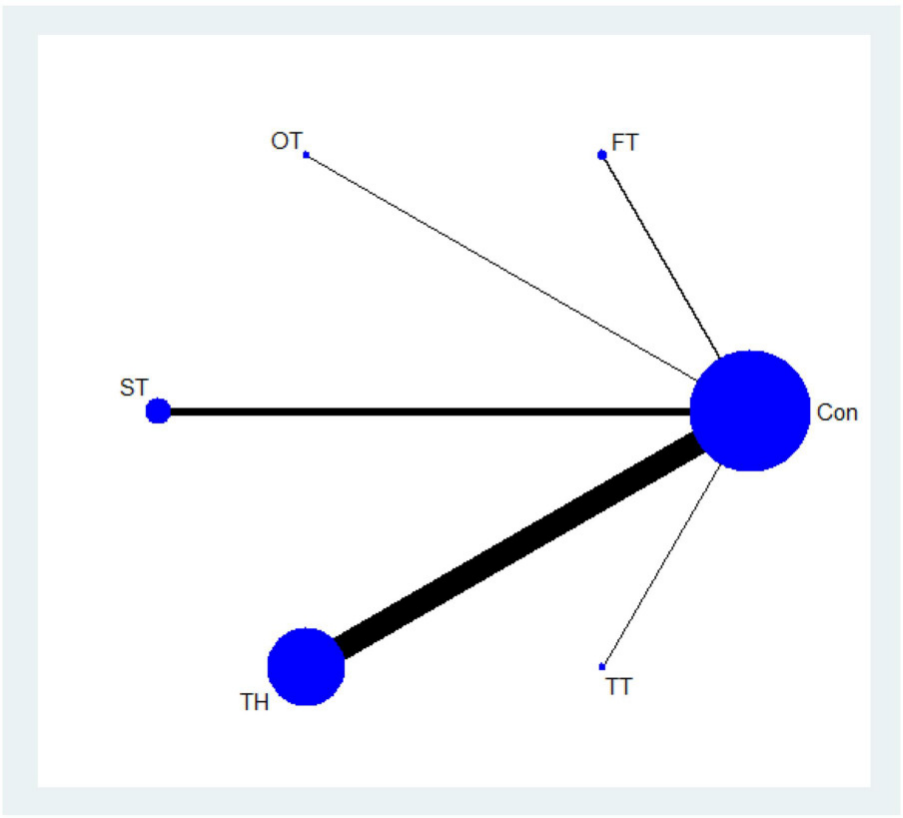
Exercise frequency network evidence diagram. OT, once per week; TT, twice per week; TH, three times per week; FT, five times per week; ST, seven times per week; Con, intervention group.

**Figure 8:**
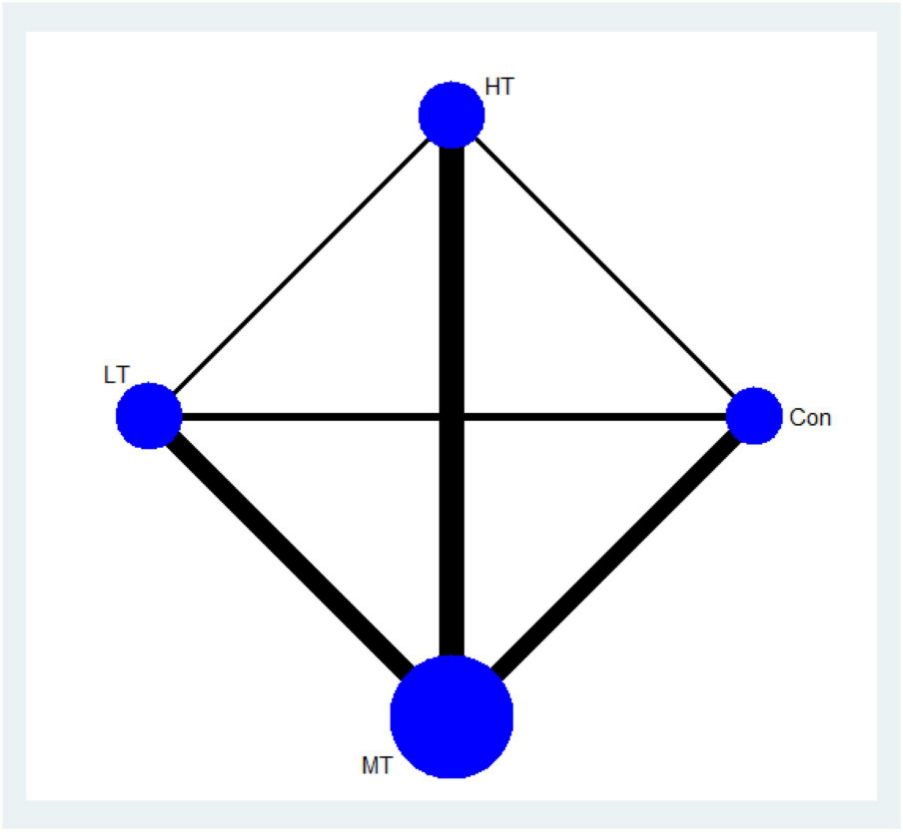
Exercise intensity network evidence diagram. LT, low intensity; MT, moderate intensity; HT, moderate-to-high intensity; Con, Intervention group.

In the network evidence diagram, each circle represents a type of exercise intervention; the more frequent the exercise intervention, the larger the circle. The thicker the line between two circles, the greater the number of studies comparing those two exercise interventions. The network evidence diagrams for exercise type, period, session duration, frequency, and intensity of the different exercise protocols are presented in Figures 4–8, respectively.

#### 3.4.2 Inconsistency test

Among the five network evidence diagrams mentioned above, the diagrams for exercise type and exercise intensity contained three and four closed loops, respectively. Global inconsistency testing showed good consistency for the pooled effects of exercise type and exercise intensity (all *P* > 0.05). The remaining interventions did not form closed loops, so inconsistency testing was not required for them. The networks for intervention period, session duration, and frequency did not form any closed loops; therefore, inconsistency could not be formally assessed using the node-splitting method. Consequently, the consistency between direct and indirect evidence in these networks could not be statistically evaluated. This also means that the probability rankings for these three components are predominantly driven by indirect evidence, which is inherently less reliable than networks supported by both direct and indirect evidence. Therefore, the SUCRA rankings for these networks are highly exploratory and should be interpreted with substantial caution.

#### 3.4.3 Results of two-by-two comparisons between elements of exercise prescription

**Figure 9:**
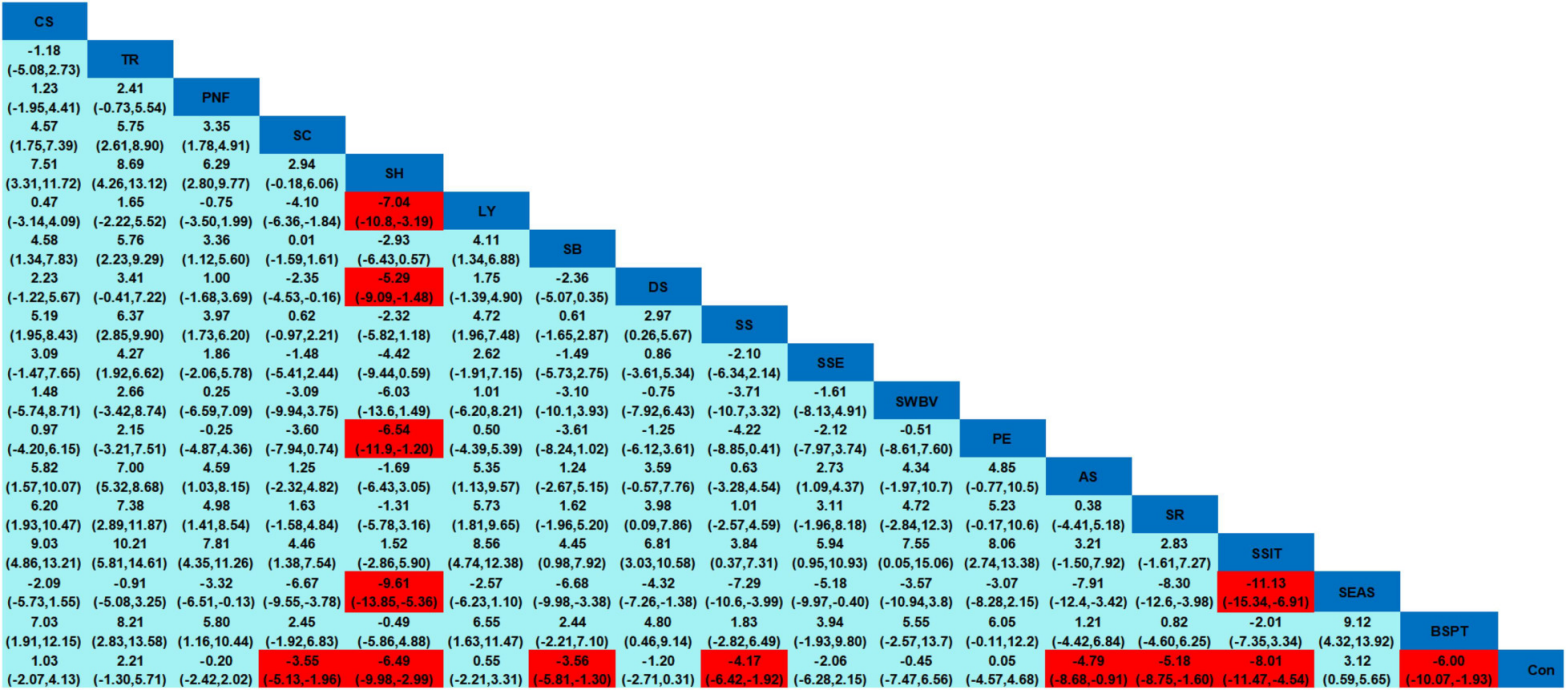
Pairwise comparison results of exercise type. CS, core stabilization exercises; TR, traditional rehabilitation exercises; PNF, proprioceptive neuromuscular facilitation; SC, Schroth; SH, schroth & hippotherapy exercises; LY, Lyon; SB, schroth & balance training; DS, daoyin spinal balance exercises; SS, schroth & sling; SSE, spinal strengthening exercises; SWBV, side-alternating whole body vibration; PE, pilates exercise; AS, active self-correction and task-oriented exercises; SR, schroth & rehabilitation exercises; SSIT, schroth & sensory integration training; SEAS, scientific exercise approach to scoliosis; BSPT, Barcelona scoliosis physical therapy school; Con, standard of care.

**Figure 10:**
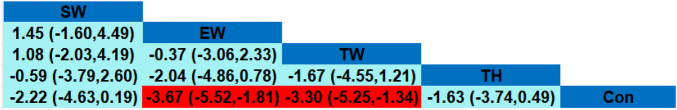
Pairwise comparison results of intervention period. SW, 6–10 Weeks; EW, 12–18 Weeks; TW, 24–26 Weeks; TH, 52 Weeks; Con, Intervention group.

**Figure 11:**
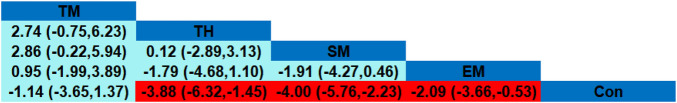
Pairwise comparison results of session duration. TM, 12–20 Min; TH, 30–45 Min; SM, 60 Min; EM, 80–90 Min; Con, Intervention group.

**Figure 12:**
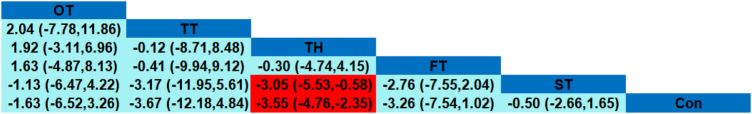
Pairwise comparison results of exercise frequency. OT, once per week; TT, twice per week; TH, three times per week; FT, five times per week; ST, seven times per week; Con, Intervention group.

**Figure 13:**
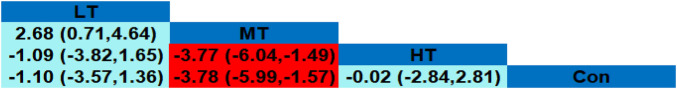
Pairwise comparison results of exercise intensity. LT, low intensity; MT, moderate intensity; HT, moderate-to-high intensity; Con, Intervention group.

1. Pairwise comparison results of exercise type. A total of 17 exercise types were included in this study. The results of the meta—analysis showed that Schroth combined with Sensory Integration Training was significantly superior to the control group, and the difference was statistically significant [MD = −8.01, 95% CI (−11.47, −4.54), *P* < 0.05]. Schroth combined with Hippotherapy Exercises was significantly superior to Lyon, and the difference was statistically significant [MD = −7.04, 95% CI (−10.8, −3.19), *P* < 0.05]. Schroth combined with Sensory Integration Training was significantly superior to the Scientific Exercise Approach to Scoliosis (SEAS), and the difference was statistically significant [MD = −11.13, 95% CI (−15.34, −6.91), *P* < 0.05]. As shown in Figure 9.
2. Pairwise comparison results of intervention period. The intervention periods of the included studies were classified into four categories: 6–10 weeks, 12–18 weeks, 24–26 weeks, and 52 weeks. The comparison results showed that exercise for 12–18 weeks was significantly superior to the control group, and the difference was statistically significant [MD = −3.67, 95% CI (−5.52, −1.81), *P* < 0.05]; exercise for 24–26 weeks was significantly superior to the control group, and the difference was statistically significant [MD = −3.30, 95% CI (−5.25, −1.34), *P* < 0.05]. As shown in Figure 10.
3. Pairwise comparison results of session duration. The session durations of the included studies were classified into four categories: 12–20 min, 30–45 min, 60 min, and 80–90 min. The comparison results showed that a session duration of 30–45 min was significantly superior to the control group, and the difference was statistically significant [MD = −3.88, 95% CI (−6.32, −1.45), *P* < 0.05]; a session duration of 60 min was significantly superior to the control group, and the difference was statistically significant [MD = −4.00, 95% CI (−5.76, −2.23), *P* < 0.05]; a session duration of 80–90 min was significantly superior to the control group, and the difference was statistically significant [MD = −2.09, 95% CI (−3.66, −0.53), *P* < 0.05]. As shown in Figure 11.
4. Pairwise comparison results of exercise frequency. The exercise frequencies of the included studies were classified into five categories: once per week, twice per week, three times per week, five times per week, and seven times per week. The comparison results showed that an exercise frequency of three times per week was significantly superior to the control group, and the difference was statistically significant [MD = −3.55, 95% CI (−4.76, −2.35), *P* < 0.05]; an exercise frequency of three times per week was significantly superior to seven times per week, and the difference was statistically significant [MD = −3.05, 95% CI (−5.53, −0.58), *P* < 0.05]. As shown in Figure 12.
5. Pairwise comparison results of exercise intensity. The exercise intensities of the included studies were classified into three categories: low intensity, moderate intensity, and moderate-to-high intensity. The comparison results showed that moderate intensity was significantly superior to the control group, and the difference was statistically significant [MD = −3.78, 95% CI (−5.99, −1.57), *P* < 0.05]; moderate intensity was significantly superior to moderate-to-high intensity, and the difference was statistically significant [MD = −3.77, 95% CI (−6.04, −1.49), *P* < 0.05]. As shown in Figure 13.

#### 3.4.4 Cumulative probabilistic ranking of interventions for each element of exercise prescription

Generally, the Cobb angle values for the efficacy of different exercise protocols in treating AIS patients are negative; therefore, for each component of the exercise protocols, the lower the cumulative probability of being the optimal intervention, the higher the probability that the intervention ranks among the better options within this specific network analysis.

**Figure 14:**
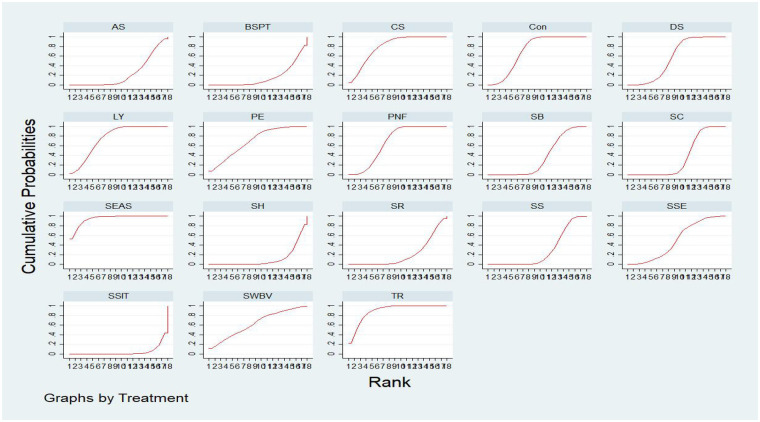
Ranking of cumulative probabilities for the optimal intervention by exercise type. CS, core stabilization exercises; TR, traditional rehabilitation exercises; PNF, proprioceptive neuromuscular facilitation; SC, Schroth; SH, schroth & hippotherapy exercises; LY, Lyon; SB, schroth & balance training; DS, daoyin spinal balance exercises; SS, schroth & sling; SSE, spinal strengthening exercises; SWBV, side-alternating whole body vibration; PE, pilates exercise; AS, active self-correction and task-oriented exercises; SR, schroth & rehabilitation exercises; SSIT, schroth & sensory integration training; SEAS, scientific exercise approach to scoliosis; BSPT, barcelona scoliosis physical therapy school; Con, standard of care.

**Figure 15:**
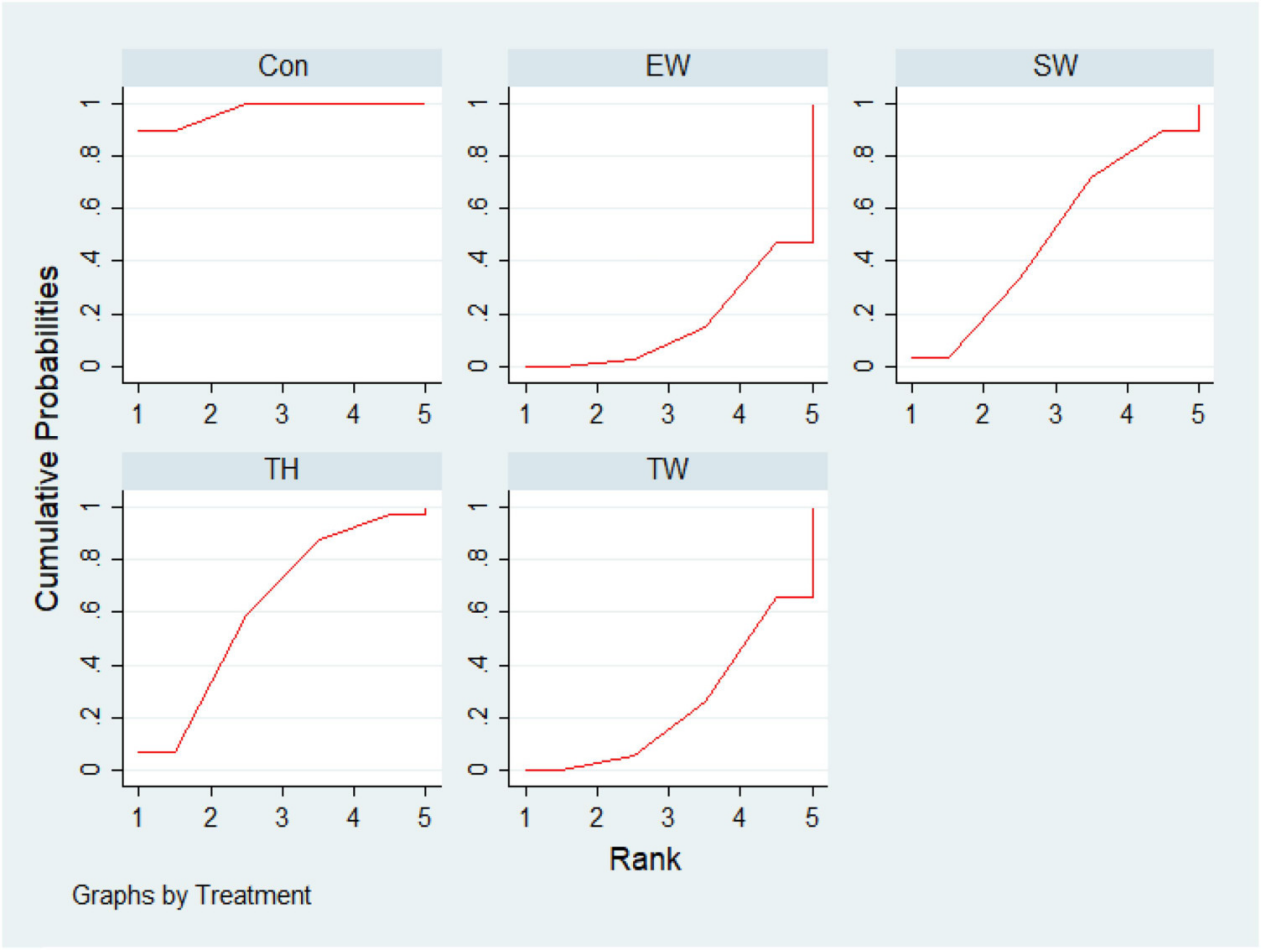
Ranking of cumulative probabilities for the optimal intervention by intervention period. SW, 6–10 Weeks; EW, 12–18 Weeks; TW, 24–26 Weeks; TH, 52 Weeks; Con, Intervention group.

**Figure 16:**
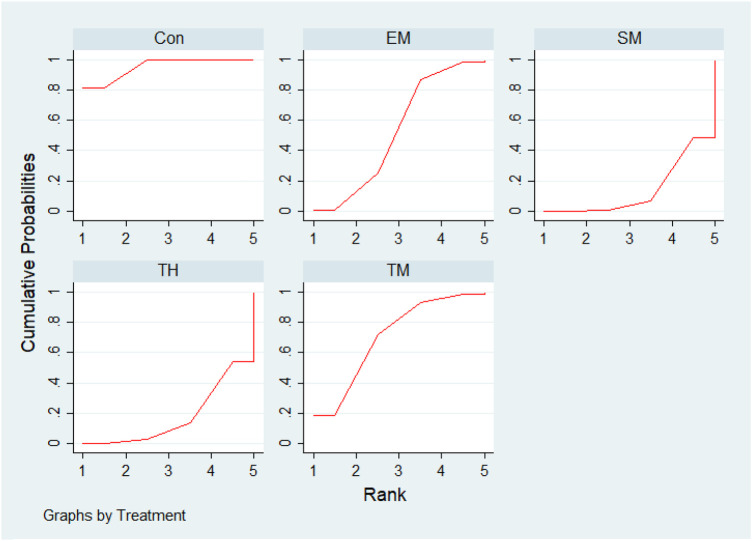
Ranking of cumulative probabilities for the optimal intervention by session duration. TM, 12–20 Min; TH, 30–45 Min; SM, 60 Min; EM, 80–90 Min; Con, intervention group.

**Figure 17:**
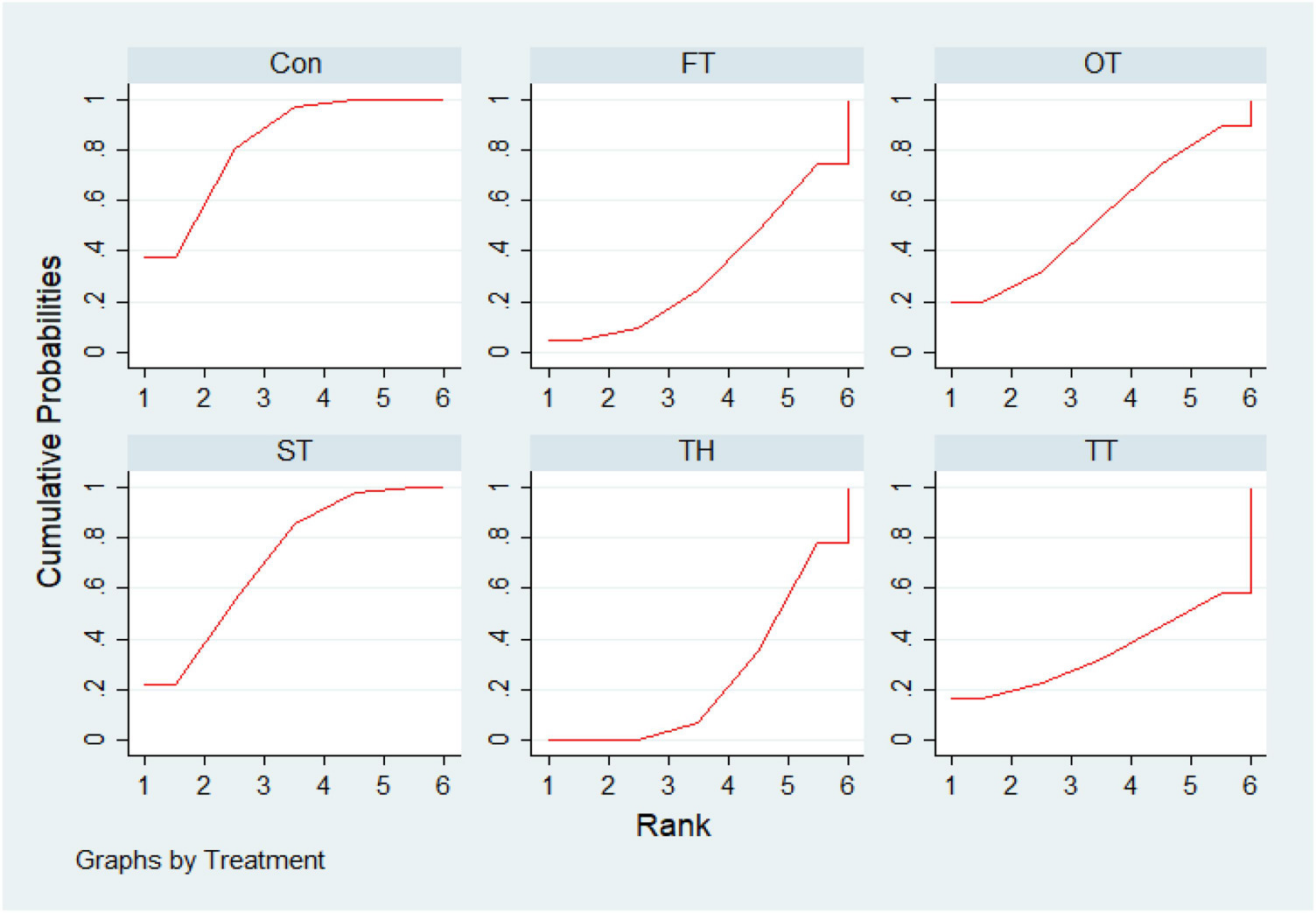
Ranking of cumulative probabilities for the optimal intervention by exercise frequency. OT, once per week; TT, twice per week; TH, three times per week; FT, five times per week; ST, seven times per week; Con, Intervention group.

**Figure 18:**
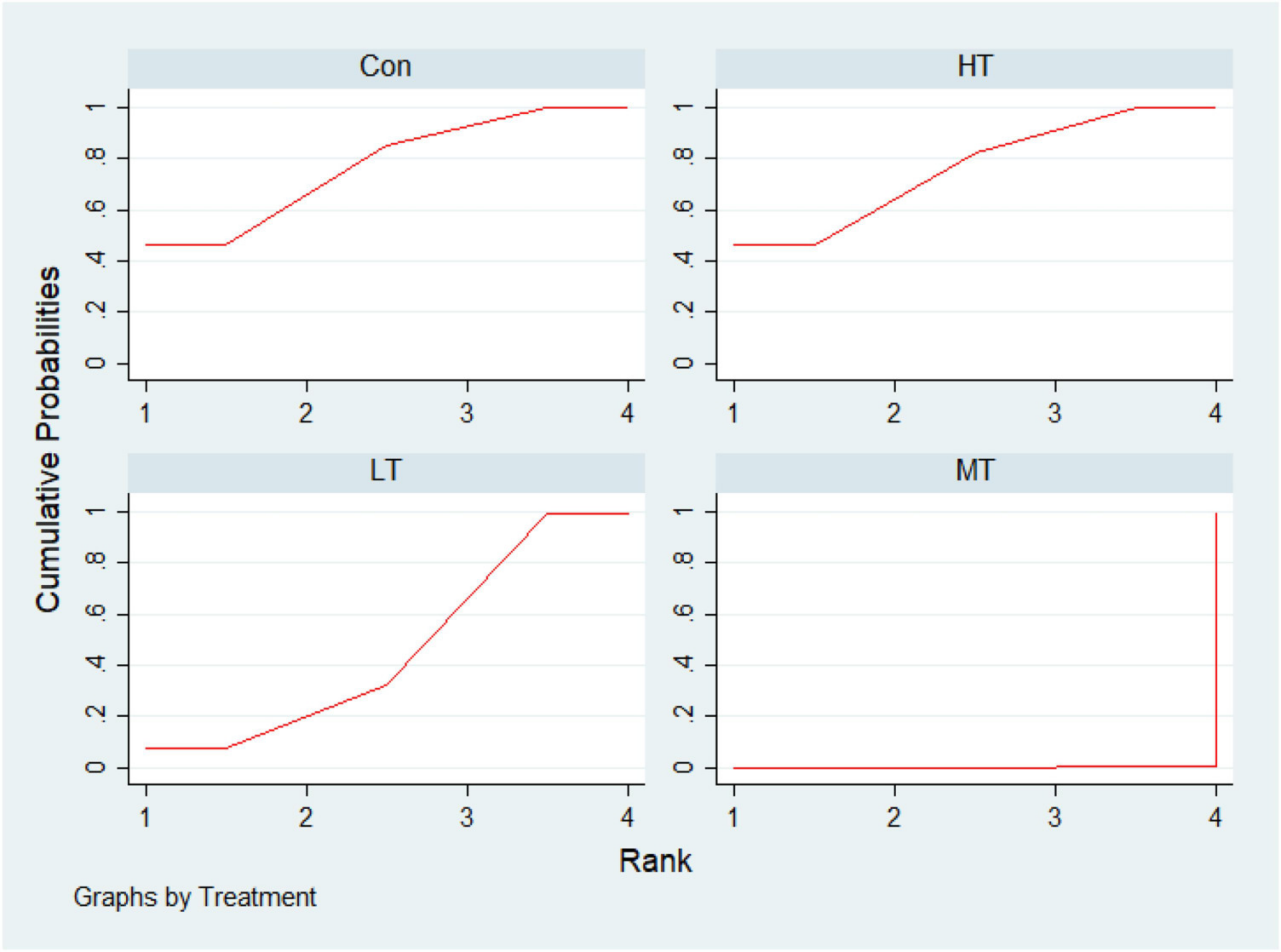
Ranking of cumulative probabilities for the optimal intervention by exercise intensity. LT, low intensity; MT, moderate intensity; HT, moderate-to-high intensity; Con, Intervention group.

1. Ranking of cumulative probabilities for each intervention by exercise type. The results of the network meta—analysis ranking the cumulative probabilities of different exercise types as each intervention showed the following order: Schroth combined with Sensory Integration Training (SUCRA = 4.3) > Schroth combined with Hippotherapy Exercises (SUCRA = 11.8) > Barcelona Scoliosis Physical Therapy School (SUCRA = 16.5) > Schroth combined with Rehabilitation Exercises (SUCRA = 21.2) > Active Self—correction and Task—oriented Exercises (SUCRA = 23.3) > Schroth combined with Sling (SUCRA = 29.0) > Schroth combined with Balance Training (SUCRA = 36.0) > Schroth (SUCRA = 36.5) > Spinal Strengthening Exercises (SUCRA = 50.4) > Daoyin Spinal Balance Exercises (SUCRA = 57.9) > Side—alternating Whole Body Vibration (SUCRA = 63.3) > Proprioceptive Neuromuscular Facilitation (SUCRA = 68.0) > Pilates Exercise (SUCRA =  70.0) > Standard of Car (SUCRA = 71.3) > Lyon (SUCRA = 76.1) > Core Stabilization Exercises (SUCRA = 80.1) > Traditional Rehabilitation Exercises (SUCRA = 89.6) > Scientific Exercise Approach to Scoliosis (SUCRA = 94.8). As shown in Figure 14.
2. Ranking of cumulative probabilities for each intervention by intervention period. The results of the network meta—analysis ranking the cumulative probabilities of different intervention periods as each intervention showed the following order: 12–18 weeks (SUCRA = 16.2) > 24–26 weeks (SUCRA = 24.3) > 6–10 weeks (SUCRA = 49.5) > 52 weeks (SUCRA = 62.9). As shown in Figure 15.
3. Ranking of cumulative probabilities for each intervention by session duration. The cumulative probability ranking for the effect of session duration on AIS patients showed the following order: 60 min (SUCRA = 13.9) > 30–45 min (SUCRA = 17.6) > 80–90 min (SUCRA = 52.5) > 12–20 min (SUCRA = 70.6). As shown in Figure 16.
4. Ranking of cumulative probabilities for each intervention by exercise frequency. The results of the network meta—analysis ranking the cumulative probabilities of different exercise frequencies as each intervention showed the following order: three times per week (SUCRA = 24.0) > five times per week (SUCRA = 32.1) > twice per week (SUCRA = 34.9) > once per week (SUCRA = 53.9) > seven times per week (SUCRA = 72.0). As shown in Figure 17.
5. Ranking of cumulative probabilities for each intervention by exercise intensity. The cumulative probability ranking for the effect of exercise intensity on AIS patients showed the following order: moderate intensity (SUCRA = 0.2) > low intensity (SUCRA = 46.6) > moderate—to—high intensity (SUCRA = 76.0). As shown in Figure 18.

### 3.5 Heterogeneity assessment and subgroup analysis

The heterogeneity test indicated substantial between-study heterogeneity (*I*^2^ = 88.9%, *Q*-test *P* < 0.05). To explore the sources of heterogeneity, a subgroup analysis was performed by classifying the exercise interventions into four overall categories: Combination therapy, Physiotherapeutic Scoliosis-Specific Exercises (PSSE), General Therapeutic Exercise (GTE), and Standard of Care (39). The results showed a statistically significant difference in pooled effect sizes across these categories (between-group difference *P* < 0.05). Among them, the Combination therapy group yielded the largest pooled effect size [MD = −6.92, 95% CI (−9.58, −4.26)], followed by the PSSE group [MD = −5.64, 95% CI (−7.29, −3.99)]. These findings suggest that the category of exercise intervention may be an important source of heterogeneity, and combined treatment approaches may offer greater benefits for improving the Cobb angle. However, given that this subgroup analysis was conducted using a pairwise meta-analytic approach, the results should be regarded as exploratory.

### 3.6 Publication bias test

**Figure 19:**
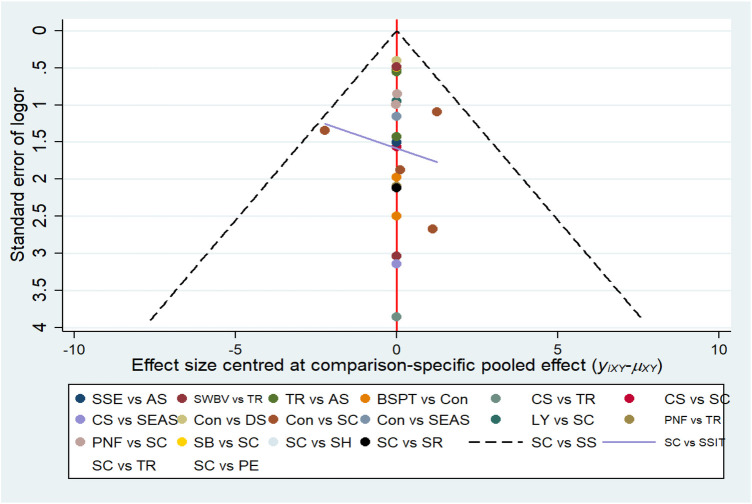
Comparison-adjusted funnel plot. CS, core stabilization exercises; TR, traditional rehabilitation exercises; PNF, proprioceptive neuromuscular facilitation; SC, Schroth; SH, schroth & hippotherapy Exercises; LY, Lyon; SB, schroth & balance Training; DS, daoyin spinal balance exercises; SS, schroth & sling; SSE, spinal strengthening exercises; SWBV, side-alternating whole body vibration; PE, pilates exercise; AS, active self-correction and task-oriented Exercises; SR, schroth & rehabilitation exercises; SSIT, schroth & sensory integration training; SEAS, scientific exercise approach to scoliosis; BSPT, Barcelona scoliosis physical therapy school; Con, Standard of Care.

An adjusted comparison funnel plot is distinct from the funnel plot used in traditional meta—analysis; it adjusts the data from different studies to a common scale, allowing them to be pooled and visually examined for publication bias. In this study, the adjusted comparison funnel plot showed good symmetry, and Egger's test yielded a *P* > 0.05, indicating that the possibility of publication bias among the included studies is low. Nevertheless, the interpretation of the results should still be approached with caution. As shown in Figure 19. It should be noted that although Egger's test yielded a *P* > 0.05, which did not reach statistical significance, the possibility of publication bias cannot be completely ruled out. First, the number of included studies was limited, which may have reduced the statistical power of Egger's test, posing a risk of a false-negative result. Second, the visual assessment of symmetry in the comparison-adjusted funnel plot involves a degree of subjectivity. Third, methodological approaches for assessing publication bias in network meta-analysis are still evolving, and no universally accepted gold standard currently exists. Therefore, the influence of publication bias on the present findings cannot be fully excluded, and the results should be interpreted with this caveat in mind.

## 4 Discussion

The etiological factors of AIS are complex and remain unclear; therefore, “early detection, early diagnosis, and early treatment” are essential for AIS patients. Although systematic reviews and meta-analyses have examined the effects of different non-surgical interventions for AIS, the present study used a network meta-analysis to explore the effects of different exercise protocol components (type, period, session duration, frequency, intensity) on AIS. The results showed that Schroth combined with sensory integration training had the highest probability of being the most effective intervention for improving the Cobb angle in AIS patients. Regarding exercise parameters, an intervention period of 12–18 weeks, a session duration of 60 min, a frequency of three times per week, and moderate-intensity exercise load ranked first in their respective probability rankings. These findings provide evidence—based support for developing individualized exercise protocols for AIS patients.

Regarding clinical relevance, the findings of this study provide a stratified evidence base for designing exercise prescriptions for AIS. Our network meta-analysis indicates that Schroth-based combination protocols, particularly Schroth combined with sensory integration training, yielded a Cobb angle improvement [MD = −8.01°, 95% CI (−11.47, −4.54)] that not only achieved statistical significance but also surpassed the widely recognized minimal clinically important differencethreshold of 5° (1). It should be noted, however, that this effect estimate is derived from only one study, making this intervention among the most clinically promising yet least evidence-supported in the current network. Therefore, its high ranking should be regarded as a clinical lead meriting priority verification, rather than an established recommendation. For AIS patients at higher risk of curve progression, this combined protocol shows encouraging therapeutic potential, but its broader clinical application requires confirmation by additional high-quality studies.

This finding differs from some previous meta-analyses, in which Schroth combined with hippotherapy exercises or the scientific exercise approach was suggested to be more effective (7, 40). In the present study, Schroth combined with sensory integration training demonstrated the greatest clinical benefit numerically, although this comparison was based on only a single study and the confidence interval was relatively wide. Several factors may explain this discrepancy. First, differences in inclusion criteria and literature search timeframes may have resulted in different evidence bases across studies; previous network meta-analyses may not have included the RCT evaluating Schroth combined with sensory integration training, thereby excluding this specific comparison from their networks. Second, differences in the categorization of exercise interventions may also contribute: some NMAs grouped interventions differently, which can influence the composition of the network and the resulting probability rankings. Beyond these methodological considerations, the different intervention targets may also play a role: Schroth exercises correct abnormal posture through sensory stimulation and mirror control; hippotherapy uses rotational pelvic movements along the longitudinal axis to achieve dynamic spinal balance; whereas sensory integration training focuses more on the central nervous system's integration of proprioceptive and vestibular information and the automation of postural control (1, 13, 41). For adolescents in a critical period of neural development (42), sensory integration training—through exercises such as one-legged stance with eyes closed, quadruped contralateral limb extension, and the dead bug exercise—promotes a higher level of postural control automation, potentially activating and remodeling neural pathways (43). This multidimensional mechanism of “active postural correction combined with central sensory integration” may explain why this combined protocol appears to produce synergistic effects that surpass those of single-modality approaches.

However, several practical factors must be carefully weighed when translating these findings into clinical recommendations. First, although Schroth combined with sensory integration training showed the largest point estimate, its 95% confidence interval was relatively wide, and the precision of its clinical benefit still needs to be confirmed by larger-sample studies. Second, this combined protocol places relatively high demands on therapist qualifications, facilities, equipment, and patient adherence, which may limit its wider implementation. In contrast, Schroth exercises applied alone, while offering only borderline clinical benefit, are relatively simple to perform, more amenable to home-based practice, and easier to maintain over the long term, making them an important initial option in resource-limited settings. Therefore, clinical decision-making should not rely solely on probability rankings or the magnitude of effect sizes; rather, it should integrate the patient's curve severity, risk of progression, neuromuscular control capacity, and healthcare accessibility, seeking an individualized balance between “optimal efficacy” and “maximal feasibility.” It should also be noted that 10 of the 24 included studies employed Schroth or its derivative approaches (e.g., BSPTS); this imbalance in the evidence base may have influenced the probability rankings and should be considered when interpreting the results.

Regarding intervention period, 12–18 weeks of exercise intervention ranked first in the probability analysis. Given that this network lacks closed loops and the ranking relies predominantly on indirect evidence, this finding should be regarded as highly exploratory. Nonetheless, it is generally consistent with the meta—analysis results of Rodrigo, Hongfang Zhou, and the intervention trial of Abdel-Aziem et al. (1, 8, 18, 48). From a neurophysiological perspective, Abdel-Aziem and colleagues suggested that a period of 12–18 weeks covers the complete neural remodeling process from “sensory input” to “motor integration” and then to “postural automation” (18). From the perspective of muscle adaptation, Ribeiro et al. considered that this period is sufficient to induce clear adaptive changes in skeletal muscles, especially the core stabilizer muscles (48). At the same time, this intervention duration offers good patient adherence and clinical feasibility, representing an optimal window that balances “physiological stimulation” with “practical implementation.”

With respect to exercise dose, the combination of a 60-minute session duration, three times per week, and moderate intensity ranked highest in the probability analysis. A session duration of 60 min showed the highest probability of being the most effective for AIS, which is consistent with existing findings (1, 44). Zhang Masen and colleagues argued that the effect of session duration is not simply linear; rather, there may be an optimal “therapeutic window” or “threshold effect.” Simply prolonging the session duration does not necessarily yield better results and may even reduce patient adherence by taking too much time. The network meta—analysis by Chen et al. also indicated that an adequate session duration is a basic prerequisite for ensuring treatment efficacy (44). Two additional considerations should be noted when interpreting the findings on session duration. First, the number of studies employing sessions shorter than 60 min was limited, and the duration varied considerably (12–50 min), which precludes a precise definition of the optimal minimum duration. Based on the available evidence, a session duration of 30–60 min may be advisable in clinical practice. Second, PSSE involves not only muscular endurance but also sustained cognitive engagement, as patients must maintain focused attention on precise postural corrections and breathing coordination. Prolonged sessions may lead to mental fatigue and diminished concentration, thereby compromising the quality of corrective movements. Therefore, when prescribing exercise sessions, clinicians should consider both the patient's physical capacity and their cognitive attentional resources, rather than simply extending session duration.

Regarding exercise frequency, three times per week showed a significant advantage in treating AIS, which aligns with the findings of Luis et al. They suggested that this frequency provides regular spinal stimulation while avoiding fatigue accumulation and injury risk (45). Concerning exercise intensity, the advantage of moderate intensity was also confirmed in studies by Park et al. (46, 47), who noted that simply pursuing high—intensity exercise training while neglecting moderate—intensity postural control and neuromuscular coordination training may be harmful for AIS patients. AIS patients often present with lengthened and weakened erector spinae muscles on the convex side and shortened and tight muscles on the concave side. Moderate-intensity isometric contractions and core stability training can selectively activate the weakened convex-side muscles, improve the coordination of deep core stabilizers such as the transversus abdominis, and enhance neuromuscular control, thereby achieving therapeutic effects for AIS. However, this finding should be interpreted with caution, as the intensity classification in the present study was derived from proxy indicators (e.g., repetitions, hold time) rather than direct physiological measures, which may have introduced misclassification bias.

When interpreting the probability rankings for the dose parameters (intervention period, session duration, and frequency), the influence of the evidence structure should be specifically considered. Unlike the networks for exercise type and exercise intensity, the networks for intervention period, session duration, and frequency did not form any closed loops, and their probability rankings are predominantly driven by indirect evidence. This means that the SUCRA rankings for these three dose parameters are highly exploratory and should be interpreted with substantial caution, as they rely predominantly on indirect evidence rather than on direct head-to-head comparisons (e.g., certain comparisons within the exercise type network). Future studies should prioritize direct comparative RCTs targeting specific dose parameters to construct more complete evidence networks and reduce reliance on indirect evidence.

In translating these findings to clinical practice, it is critical to recognize that the SUCRA rankings provide an estimate of the probability of being the best intervention within this specific network, and should not be interpreted as a definitive hierarchy of clinical superiority. The results suggest that protocols combining Schroth with sensory integration training, delivered at moderate intensity, three times per week, 60 min per session, for a total period of 12–18 weeks, are likely to be effective and merit further investigation. However, a truly evidence-based clinical recommendation requires the integration of these comparative efficacy data with a careful assessment of the individual patient's curve severity, tolerance, preferences, and access to specific treatment resources. It should be emphasized that these probability rankings serve as a general guide for protocol selection, and do not imply that a single “optimal” protocol is suitable for all patients. Exercise prescriptions must be tailored to the individual patient's curve characteristics, skeletal maturity, physical capacity, cognitive engagement, and personal preferences. Special attention should be paid to intervention factors that may exceed the therapeutic threshold, such as a session duration of 90 min and moderate-to-high exercise intensity.

The findings of this study should also be interpreted in light of the methodological quality of the included trials. Several included studies did not adequately report allocation concealment and blinding procedures. Given that Cobb angle measurement is subject to some degree of observer variability, if outcome assessors were aware of group allocation, detection bias may have been introduced, and the direction of such bias typically favors an overestimation of the treatment effect. Consequently, the effect sizes reported for some exercise protocols relative to the control group may be somewhat inflated. Future studies should implement rigorous assessor blinding, with Cobb angle measurements performed by a third party independent of the intervention team, to minimize the risk of detection bias.

Regarding publication bias, although Egger's test yielded a non-significant *P* value of 0.1803 and the comparison-adjusted funnel plot showed reasonable symmetry, these findings should be interpreted with substantial caution. The statistical power of Egger's test is known to be limited in the context of network meta-analysis, particularly when the number of included studies is modest, which increases the risk of a false-negative result. Therefore, the present assessment relies more heavily on the visual evaluation of the comparison-adjusted funnel plot, which, while suggestive of symmetry, does not definitively rule out publication bias. The possibility that small studies with null or negative findings remain unpublished cannot be excluded, and this should be borne in mind when interpreting the overall evidence synthesis.

## 5 Limitations

The present study has the following limitations: (1) Several included studies did not adequately report allocation concealment and blinding procedures, and the lack of blinded outcome assessment may have introduced detection bias given the susceptibility of Cobb angle measurement to observer variability. (2) The classification of exercise intensity was derived indirectly from training dose parameters rather than from direct physiological indicators, which may have introduced misclassification bias. The finding regarding moderate intensity should therefore be regarded as exploratory. (3) Only the networks for exercise type and exercise intensity contained closed loops and underwent inconsistency testing. For the networks of intervention period, session duration, and frequency, however, all control groups were defined as a single category and their effect sizes were merged, resulting in star-shaped or tree-like structures. This approach not only precluded statistical verification of evidence consistency but also potentially introduced selection bias and undermined the randomization structure of some original studies due to the merging of control groups from different sources, thereby affecting the reliability of indirect comparisons. (4) The included studies were predominantly conducted in Asian populations. Differences across populations in body habitus, cultural attitudes, and healthcare delivery models may limit generalizability to non-Asian populations. (5) Although publication bias was not detected, the risk of underestimation remains. The restriction of the literature search to English and Chinese databases may have introduced language bias. (6) Most of the included studies assessed Cobb angle immediately post-intervention, and long-term follow-up data (e.g., 1 to 2 years after intervention) are lacking. Given that AIS is a progressive, growth-related condition, the durability of exercise effects beyond the intervention period remains unknown. This limitation reflects a gap in the primary literature and should be addressed by future trials incorporating extended follow-up assessments. (7) The content of control conditions varied across included studies, which may have introduced heterogeneity in the comparative baseline. (8) Adherence to exercise interventions was not consistently reported across the included studies. Variations in patient compliance may have influenced the pooled effect estimates, particularly for longer-duration protocols. Additionally, one included study had a mean age of 8.9 years; the inclusion of participants near the boundary of early-onset scoliosis may have introduced some etiological heterogeneity.

## 6 Conclusion

In conclusion, limited evidence from this network meta-analysis suggests that, among the protocols compared, Schroth combined with sensory integration training, delivered at moderate intensity, three times per week, 60 min per session, for 12–18 weeks, has the highest probability of being the most effective protocol for improving the Cobb angle in adolescent idiopathic scoliosis. However, this finding represents a probabilistic ranking rather than definitive proof of clinical superiority, and each component of this protocol (exercise type, period, session duration, frequency, and intensity) is supported by a limited number of studies within its respective network, which further constrains the strength of the conclusion. Future well-designed, head-to-head randomized controlled trials with rigorous blinding procedures and more diverse racial/ethnic populations are urgently needed to confirm these comparative effects. Any clinical application should be highly individualized.

## Data Availability

The original contributions presented in the study are included in the article/Supplementary Material, further inquiries can be directed to the corresponding author.

## References

[1] Zhang MS Hu QQ Cui J. Effect of Schroth therapy on adolescent idiopathic scoliosis: a meta-analysis. Chin J Evid Based Med. (2025) 25(9):1033–8. 10.7507/1672-2531.202502064

[2] Wang Z Zhu W Li G Guo X. Comparative efficacy of six types of scoliosis-specific exercises on adolescent idiopathic scoliosis: a systematic review and network meta-analysis. BMC Musculoskelet Disord. (2024) 25(1):1070. 10.1186/s12891-024-08223-1 39725973 PMC11670383

[3] Chang Y Xia Y Sun YD. Effectiveness of different specific exercise therapies in treatment of adolescent idiopathic scoliosis: a network meta-analysis. Chin J Tissue Eng Res. (2024) 28(36):5899–904. 10.12307/2024.692

[4] Zhang W Bo SM Wang T. Efficacy of exercise intervention in adolescents with mild to moderate idiopathic scoliosis. A meta-analysis.Chin J Evid Based Med. (2022) 22(8):896–900. 10.7507/1672-2531.202202039

[5] Jiang Y Peng H Song Y Huang L Chen H Li P. Evaluating exercise therapies in adolescent idiopathic scoliosis: a systematic review with Bayesian network meta-analysis. PeerJ. (2025) 13:19175. 10.7717/peerj.19175 PMC11967429 40183057

[6] Liu X Wang Y Liu M Zhang Y Wu Q Wang Q. The efficacy of core stabilization exercise in mild and moderate adolescent idiopathic scoliosis: a systematic review and meta-analysis. J Orthop Surg Res. (2025) 20(1):214. 10.1186/s13018-025-05612-7 40016756 PMC11869405

[7] Liu JL Abuduwupuer H Bai Z. Efficacy of different nonsurgical treatments for adolescent idiopathic scoliosis: a systematic review and network meta-analysis. Chin J Tissue Eng Res. (2026) 30(9):2370–9. 10.12307/2026.651

[8] Zhu H Li CJ Tian Z. Effect of exercise therapy on adolescent idiopathic scoliosis in mild to moderate: a systematic review and network meta-analysis. Front Med (Lausanne). (2025) 12:1708970. 10.3389/fmed.2025.1708970 41357506 PMC12675335

[9] Ren J Wang S Li M Zhou X Wen Y Wen Z. Comparative efficacy of conservative interventions for adolescent idiopathic scoliosis: a systematic review and network meta-analysis of randomized controlled trials. Syst Rev. (2025) 14(1):156. 10.1186/s13643-025-02893-1 40739527 PMC12309149

[10] Wu ZJ Wang ZY Song YLQ. The meta-analysis about the improving effects of different exercise prescription in patients with type 2 diabetes. China Sport Sci Technol. (2017) 53(1):73–82. 10.16470/j.csst.201701

[11] Chen Y Zhang ZD Zhu QH. The effect of an exercise intervention on adolescent idiopathic scoliosis: a network meta-analysis. J Orthop Surg Res. (2023) 18(1):655. 10.1186/s13018-023-04137-1 37667353 PMC10476432

[12] Li X Tang K Zhang Y Tang L Wei K Tang M. Effect of Tai Chi on the pain intensity or disability of patients with chronic low back pain: a systematic review and meta-analysis. Front Sports Act Living. (2026) 8:1676045. 10.3389/fspor.2026.1676045 41757298 PMC12935452

[13] Guan Y Zhao B Fan Y Li Y Wang H. Protective and risk physical activities for adolescent idiopathic scoliosis: a systematic review identifying one-hour daily activity threshold and Chinese school-based prevention framework. Front Sports Act Living. (2025) 7:1644314. 10.3389/fspor.2025.1644314 40969977 PMC12441802

[14] Gür G Ayhan C Yakut Y. The effectiveness of core stabilization exercise in adolescent idiopathic scoliosis: a randomized controlled trial. Prosthet Orthot Int. (2017) 41(3):303–10. 10.1177/0309364616664151 27625122

[15] Won S Oh D-K Shen M. An 18-month follow-up study on the effect of a neuromuscular stabilization technique on Cobb's angle in adolescent idiopathic scoliosis: a single-blind, age-matched controlled trial. J Back Musculoskelet Rehabil. (2021) 34(1):87–93. 10.3233/BMR-191559 32986652

[16] Mohamed RA Yousef AM. Impact of Schroth three-dimensional vs. Proprioceptive neuromuscular facilitation techniques in adolescent idiopathic scoliosis: a randomized controlled study. Eur Rev Med Pharmacol Sci. (2021) 25(24):7717–25. 10.26355/eurrev_202112_27618 34982433

[17] Zapata KA Dieckmann RJ Hresko MT Sponseller PD Vitale MG Glassman SD. A United States multi-site randomized control trial of Schroth-based therapy in adolescents with mild idiopathic scoliosis. Spine Deform. (2023) 11(4):861–9. 10.1007/s43390-023-00665-2 36807105

[18] Abdel-aziem AA Abdelraouf OR Ghally SA Dahlawi HA Radwan RE. A 10-week program of combined hippotherapy and Scroth's exercises improves balance and postural asymmetries in adolescence idiopathic scoliosis: a randomized controlled study. Children (Basel, Switzerland). (2021) 9(1):23. 10.3390/children9010023 35053648 PMC8774272

[19] Büyükturan Ö Kaya MH Alkan H Büyükturan B Erbahçeci F, Comparison of the efficacy of Schroth and Lyon exercise treatment techniques in adolescent idiopathic scoliosis: a randomized controlled, assessor and statistician blinded study. Musculoskelet Sci Pract. (2024) 72: 102952 10.1016/j.msksp.2024.102952 38631273

[20] Kaya MH Büyükturan Ö Büyükturan B Alkan H Erbahçeci F. Comparison of the efficacy of the Schroth method and proprioceptive neuromuscular facilitation technique in adolescent idiopathic scoliosis: a randomized controlled, single-blinded study. J Bodyw Mov Ther. (2025) 43:35–41. 10.1016/j.jbmt.2025.04.003 40483147

[21] Shen X Yang Z Zhang P Xu Y Wang J. Effects of balance training combined with Schroth therapy on adolescents with mild idiopathic scoliosis: a six-week randomized controlled trial. J Back Musculoskelet Rehabil. (2023) 36(6):1365–73. 10.3233/BMR-220383 37458026

[22] Hui W Jinyuan X Zhong J Shuliang Y Hongquan S Xitao N. Effect of a traditional Chinese medicine combined therapy on adolescent idiopathic scoliosis: a randomized controlled trial. J Tradit Chin Med. (2015) 35(5):514–9. 10.1016/s0254-6272(15)30133-3 26591680

[23] Zhang P Shen X Zhang L Wang S Wu Q. Effect of sling exercise combined with Schroth therapy on adolescents with mild idiopathic scoliosis: a twelve-week randomized controlled trial. J Back Musculoskelet Rehabil. (2024) 37(2):379–88. 10.3233/BMR-230102 38043003

[24] Dimitrijević V Rašković B Popović MP Viduka D Nikolić S Jevtić N. Treatment of adolescent idiopathic scoliosis with the conservative Schroth method: a randomized controlled trial. Healthcare (Basel, Switzerland). (2025) 13(6):688. 10.3390/healthcare13060688 40150538 PMC11942212

[25] Kumar A Kumar S Sharma V. Efficacy of task oriented exercise program based on ergonomics on cobb’s angle and pulmonary function improvement in adolescent idiopathic scoliosis- A randomized control trial. J Clin Diagn Res. (2017) 11(8):YC01–4. 10.7860/JCDR/2017/27497.10335 28969262 PMC5620903

[26] Langensiepen S Stark C Sobottke R Semler O Franklin J Schraeder M. Home-based vibration assisted exercise as a new treatment option for scoliosis—a randomised controlled trial. J Musculoskelet Neuronal Interact. (2017) 17(4):259–67. 29199184 PMC5749031

[27] Kim G Hwang PB. Effects of Schroth and pilates exercises on the Cobb angle and weight distribution of patients with scoliosis. J Phys Ther Sci. (2016) 28(3):1012–5. 10.1589/jpts.28.1012 27134403 PMC4842415

[28] Kyrkousis A Iakovidis P Chatziprodromidou IP Lytras D Kasimis K Apostolou T. Effects of a long-term supervised Schroth exercise program on the severity of scoliosis and quality of life in individuals with adolescent idiopathic scoliosis: a randomized clinical trial study. Medicina (Kaunas, Lithuania). (2024) 60(10):1637. 10.3390/medicina60101637 39459424 PMC11509648

[29] Monticone M Ambrosini E Cazzaniga D Rocca B Ferrante S. Active self-correction and task-oriented exercises reduce spinal deformity and improve quality of life in subjects with mild adolescent idiopathic scoliosis. Results of a randomised controlled trial. Eur Spine J. (2014) 23(6):1204–14. 10.1007/s00586-014-3241-y 24682356

[30] Schreiber S Parent EC Khodayari Moez E Hedden DM Hill DL Moreau M. Schroth physiotherapeutic scoliosis-specific exercises added to the standard of care lead to better cobb angle outcomes in adolescents with idiopathic scoliosis—an assessor and statistician blinded randomized controlled trial. PLoS One. (2016) 11(12):e0168746. 10.1371/journal.pone.0168746 28033399 PMC5198985

[31] Kocaman H Bek N Kaya MH Büyükturan B Yetiş M Büyükturan Ö. The effectiveness of two different exercise approaches in adolescent idiopathic scoliosis: a single-blind, randomized-controlled trial. PLoS One. (2021) 16(4):e0249492. 10.1371/journal.pone.0249492 33857180 PMC8049223

[32] Zhang Y Chai T Weng H Liu Y. Pelvic rotation correction combined with Schroth exercises for pelvic and spinal deformities in mild adolescent idiopathic scoliosis: a randomized controlled trial. PLoS One. (2024) 19(7):e0307955. 10.1371/journal.pone.0307955 39078854 PMC11288462

[33] Lee S-G Park H-S Çolak TK Yoon J-H Oh J-K. Preliminary effects of bracing and the Schroth method on adolescent idiopathic scoliosis with low risser grade: a randomized controlled trial. J Back Musculoskelet Rehabil. (2026) 39(2):564–75. 10.1177/10538127251382688 41032672

[34] Liang W Zhou Z Lv Y Zhou W Wang H. Effect of sensory integration training combined with Schroth therapy on adolescents with mild idiopathic scoliosis: a 12-week randomized controlled trial. Eur J Med Res. (2025) 31(1):59. 10.1186/s40001-025-03607-y 41354983 PMC12797335

[35] Zapata KA Sucato DJ Jo C-H. Physical therapy scoliosis-specific exercises may reduce curve progression in mild adolescent idiopathic scoliosis curves. Pediatr Phys Ther. (2019) 31(3):280–5. 10.1097/PEP.0000000000000621 31220013

[36] Yagci G Yakut Y. Core stabilization exercises versus scoliosis-specific exercises in moderate idiopathic scoliosis treatment. Prosthet Orthot Int. (2019) 43(3):301–8. 10.1177/0309364618820144 30628526

[37] Zheng Y Dang Y Yang Y Li H Zhang L Lou EHM. Whether orthotic management and exercise are equally effective to the patients with adolescent idiopathic scoliosis in mainland China? A randomized controlled trial study. Spine. (2018) 43(9):E494–503. 10.1097/BRS.0000000000002412 28885287

[38] Lin Y Yu D Chen X Chen P Chen N Shao B. Effects of proprioceptive exercise for knee osteoarthritis: a systematic review and meta-analysis. Front Rehabil Sci. (2025) 6:1596966. 10.3389/fresc.2025.1596966 40631343 PMC12234485

[39] Romano M Minozzi S Bettany-Saltikov J Zaina F Chockalingam N Kotwicki T. Therapeutic exercises for idiopathic scoliosis in adolescents. Cochrane Database Syst Rev. (2024) 2(4):10–32. 10.1002/14651858.cd007837.pub3 PMC10900302 38415871

[40] Dong H You M Li Y Wang B Huang H. Physiotherapeutic scoliosis-specific exercise for the treatment of adolescent idiopathic scoliosis: a systematic review and network meta-analysis. Am J Phys Med Rehabil. (2024) 104(1):14–25. 10.1097/PHM.0000000000002524 38726971

[41] Brellenthin AG Lanningham-Foster LM Kohut ML Li Y Church TS Blair SN. Comparison of the cardiovascular benefits of resistance, aerobic, and combined exercise (CardioRACE): rationale, design, and methods. Am Heart J. (2019) 217:101–11. 10.1016/j.ahj.2019.08.008 31520895 PMC6861681

[42] Blakemore SJ Choudhury S. Development of the adolescent brain: implications for executive function and social cognition. J Child Psychol Psychiatry. (2006) 47(4):296–312. 10.1111/j.1469-7610.2006.01611.x 16492261

[43] Taubert M Draganski B Anwander A Müller K Horstmann A Villringer A. Dynamic properties of human brain structure: learning-related changes in cortical areas and associated fiber connections. J Neurosci. (2010) 30(35):11670–7. 10.1523/JNEUROSCI.2567-10.2010 20810887 PMC6633410

[44] Chen J Xu T Zhou J Han B Wu Q Jin W. The superiority of Schroth exercise combined brace treatment for mild-to-moderate adolescent idiopathic scoliosis: a systematic review and network meta-analysis. World Neurosurg. (2024) 186:184–196.e9. 10.1016/j.wneu.2024.03.103 38531472

[45] Ceballos-Laita L Carrasco-Uribarren A Cabanillas-Barea S Pérez-Guillén S Pardos-Aguilella P Jiménez Del Barrio S. The effectiveness of Schroth method in Cobb angle, quality of life and trunk rotation angle in adolescent idiopathic scoliosis: a systematic review and meta-analysis. Eur J Phys Rehabil Med. (2023) 59(2):228–36. 10.23736/S1973-9087.23.07654-2 36692412 PMC10170402

[46] Park J So W-Y. The effect of the Schroth rehabilitation exercise program on spinal and feet alignment in adolescent patients with idiopathic scoliosis: a pilot study. Healthcare (Basel, Switzerland). (2022) 10(2):398. 10.3390/healthcare10020398 35207011 PMC8871911

[47] MacIntosh BR Murias JM Keir DA Weir JM. What is moderate to vigorous exercise intensity? Front Physiol. (2021) 12:682233. 10.3389/fphys.2021.682233 34630133 PMC8493117

[48] Andrade RM Ribeiro AP Ferreira MEC Piras LC De Moura Partika ML Junior NC. Impact of therapeutic exercises versus general conservative modalities and brace on the progression of adolescent idiopathic scoliosis: systematic review and meta-analysis. Arch Phys Med Rehabil. (2025) 106(12):1874–85. 10.1016/j.apmr.2025.06.021 40712865

