# Supplementary material for: Comparative efficacy of different scoliosis-specific exercise protocols on cobb angle in adolescent idiopathic scoliosis: a network meta-analysis
Source: Front Sports Act Living. 2026 Jun 25;8:1847841. doi: 10.3389/fspor.2026.1847841 (PMC13346065; doi:10.3389/fspor.2026.1847841)
Supplement: Supplementary file 1 [file Supplementaryfile1.docx]

Supplementary Material

TABLE 1 Search strategy

((("Scoliosis"[Mesh]) OR (Adolescent Idiopathic Scoliosis[Title/Abstract])) AND (("Exercise"[Mesh]) OR (((((((((((((((((((((((((Exercises[Title/Abstract]) OR (Exercise, Physical[Title/Abstract])) OR (Schroth Exercise[Title/Abstract])) OR (Lyon Exercise[Title/Abstract])) OR (Pilates Exercises[Title/Abstract])) OR (Scientific Exercise，Approach to Scoliosis[Title/Abstract])) OR (Core Stabilization Exercise[Title/Abstract])) OR (Barcelona Scoliosis Physical Therapy School[Title/Abstract])) OR (Physiotherapy Scoliosisspecific Exercise[Title/Abstract])) OR (Functional Individual Therapy of Scoliosis[Title/Abstract])) OR (Yoga[Title/Abstract])) OR (Proprioceptive Neuromuscular Facilitation[Title/Abstract])) OR (Combination Therapy[Title/Abstract])) OR (Schroth Exercise and Hippotherapy Training[Title/Abstract])) OR (Physiotherapy Scoliosis-Specific Exercise[Title/Abstract])) OR (Schroth Exercise and Sling Training[Title/Abstract])) OR (Schroth Exercise and Balance Training[Title/Abstract])) OR (Pelvic Rotation Correction and Schroth Exercise[Title/Abstract])) OR (Daoyin Spinal Balance Exercises[Title/Abstract])) OR (Active Self-correction and Task-oriented Exercises[Title/Abstract])) OR (Side-alternating Whole Body Vibration[Title/Abstract])) OR (Schroth and Sensory Integration Training[Title/Abstract])) OR (Spinal Strengthening Exercises[Title/Abstract])) OR (Activity, Physical[Title/Abstract])) OR (Physical Activities[Title/Abstract])))) AND (Randomized controlled trial[publication type] or randomized[title/abstract] or placebo[title/abstract])

FIGURE 1 PRISMA flow diagram of the study process.

Records identified from*:

Databases (n =471 )

PubMed (n =104 ),

Embase (n =94 ),

Web of Science (n =61 ),

CNKI (n =44 ),

Wan Fang (n =79 ),

VIP(n =89 )

Records removed *before screening*:

Duplicate records removed (n =237 )

Records screened

(n =234 )

Records excluded**

(n =122 )

Reports sought for retrieval

(n = 112)

Reports not retrieved

(n =73 )

Reports assessed for eligibility

(n =39 )

Reports excluded:

Inconsistent research design(N=5)

Inconsistent intervention measures(N=3)

Inconsistent research subjects(N=1)

Inconsistent outcome indicators(N=6)

Reports of included studies

(n = 24)

**Identification of studies via databases and registers**

**Identification**

**Screening**

**Included**


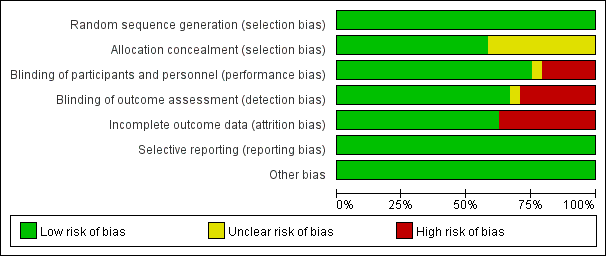


FIGURE 2 Overall bias risk diagram.


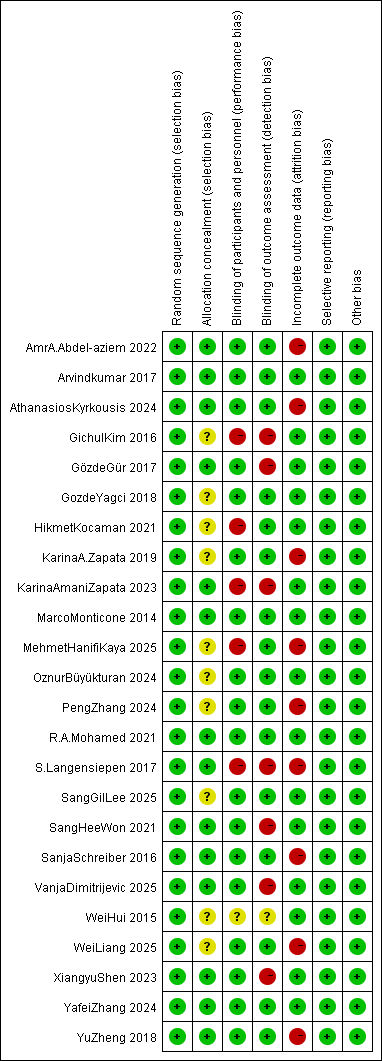


FIGURE 3 Bias risk diagram for each item.


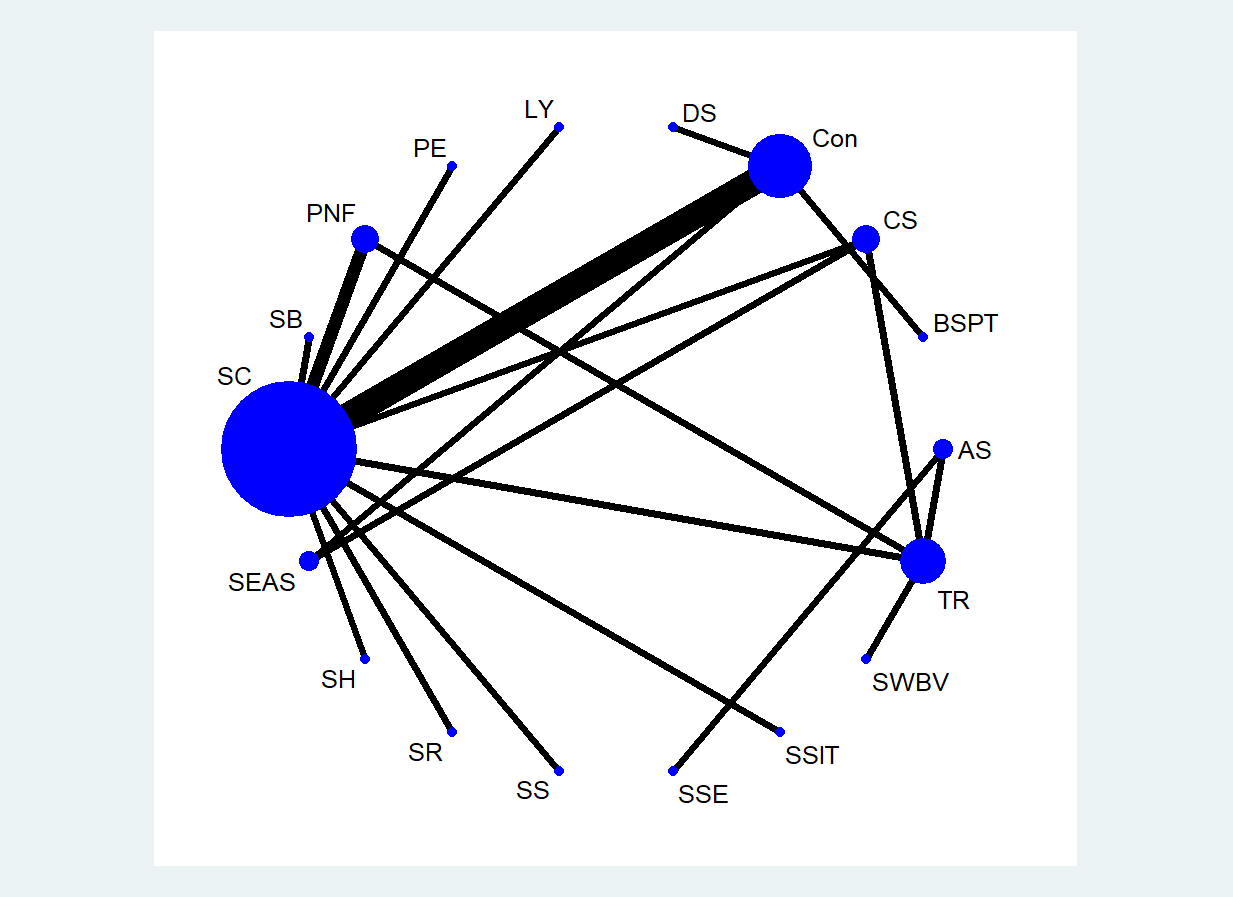


FIGURE 4. Exercise type network evidence diagram. CS:Core Stabilization Exercises; TR:Traditional Rehabilitation Exercises; PNF:Proprioceptive Neuromuscular Facilitation; SC：Schroth；SH:Schroth & Hippotherapy Exercises; LY:Lyon; SB:Schroth & Balance Training; DS:Daoyin Spinal Balance Exercises; SS:Schroth & Sling; SSE:Spinal Strengthening Exercises; SWBV:Side-alternating Whole Body Vibration; PE:Pilates Exercise; AS:Active Self-correction and Task-oriented Exercises; SR:Schroth & Rehabilitation Exercises; SSIT:Schroth & Sensory Integration Training; SEAS:Scientific Exercise Approach to Scoliosis; BSPT:Barcelona Scoliosis Physical Therapy School; Con:Standard of Care.


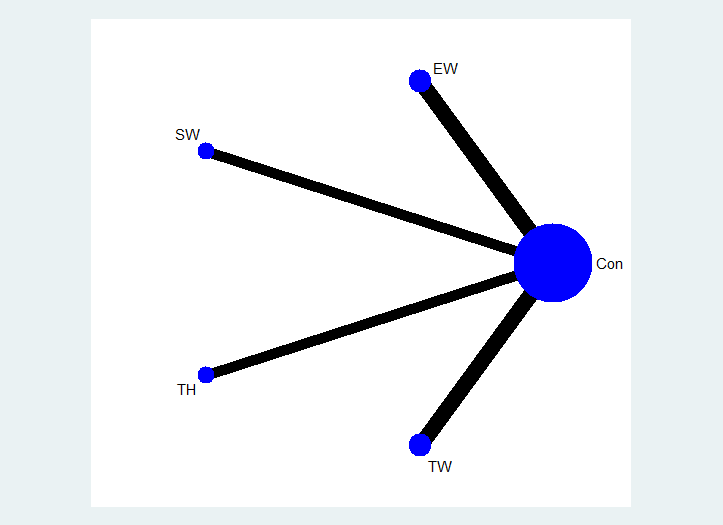


FIGURE 5. Intervention period network evidence diagram. SW:6-10 Weeks; EW：12-18 Weeks; TW：24-26 Weeks; TH：52 Weeks; Con:Intervention group.


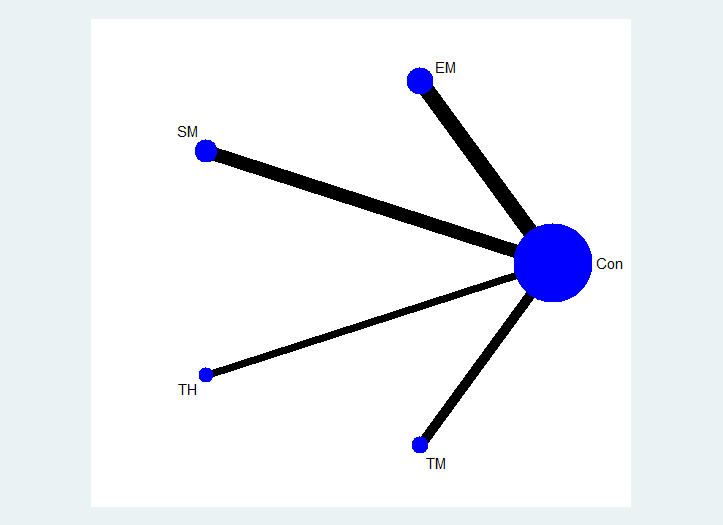


FIGURE 6. Session duration network evidence diagram. TM: 12-20 Min；TH：30-45 Min；SM：60 Min ；EM：80-90 Min ；Con:Intervention group.


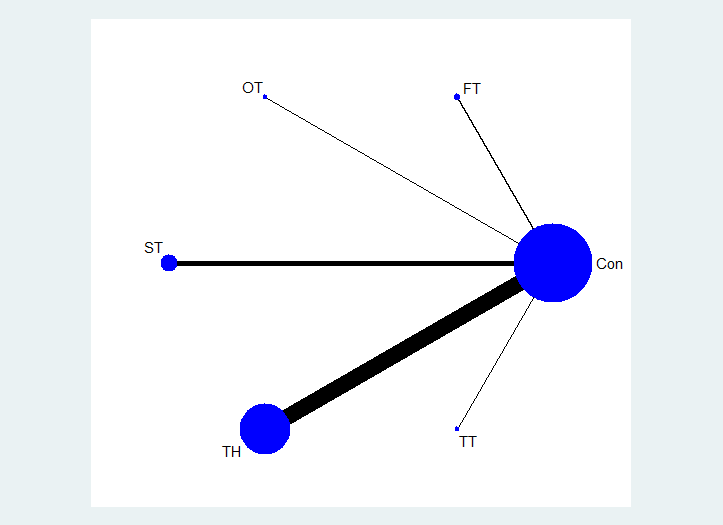


FIGURE 7. Exercise frequency network evidence diagram. OT：once per week; TT：twice per week; TH：three times per week; FT：five times per week; ST：seven times per week; Con:Intervention group.

**
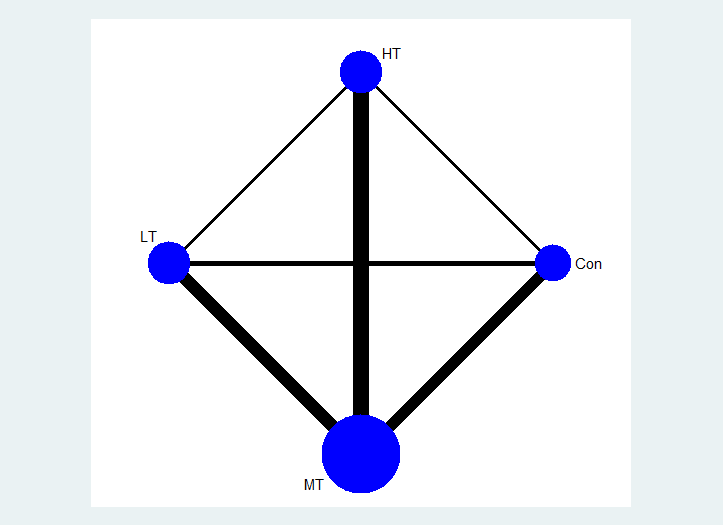
**

FIGURE 8. Exercise intensity network evidence diagram. LT:low intensity；MT：moderate intensity；HT： moderate-to-high intensity；Con:Intervention group.


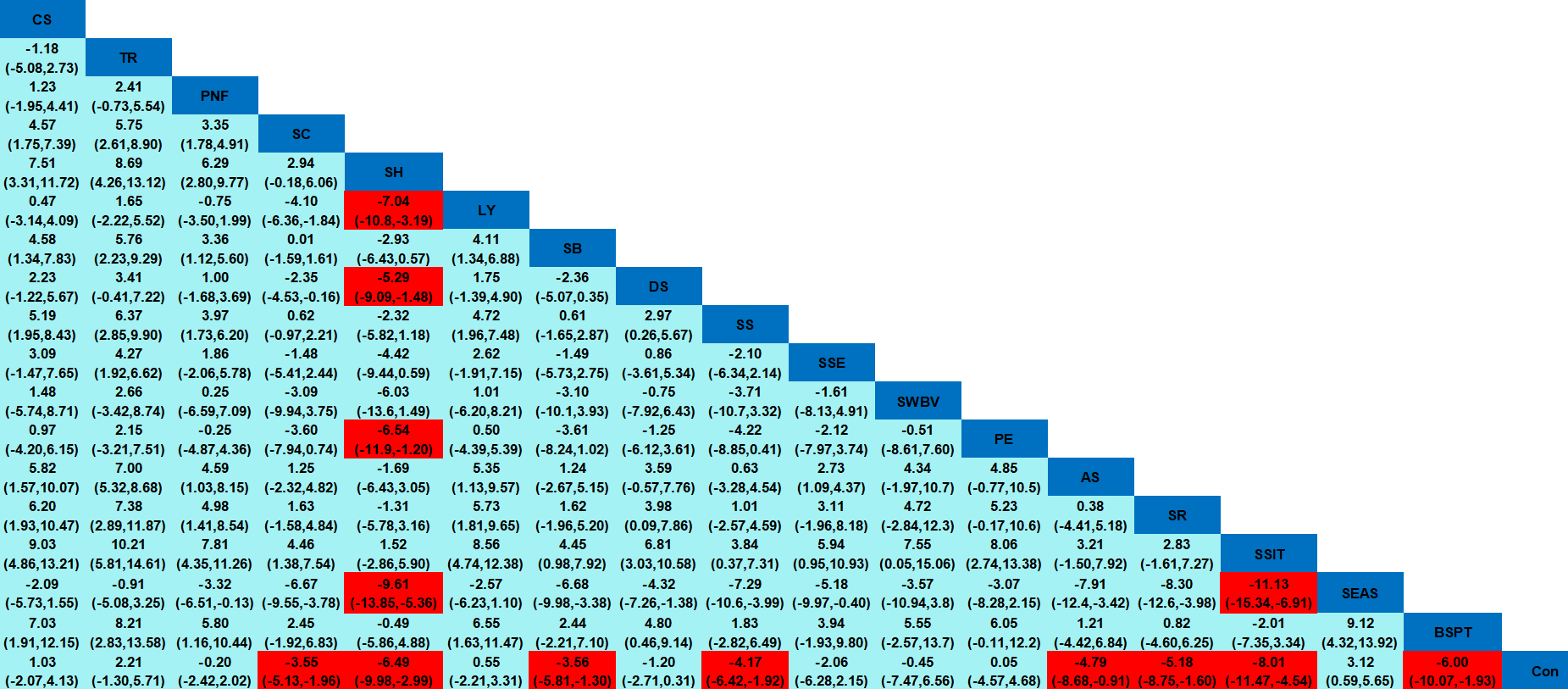


FIGURE 9. Pairwise comparison results of exercise type . CS:Core Stabilization Exercises; TR:Traditional Rehabilitation Exercises; PNF:Proprioceptive Neuromuscular Facilitation; SC：Schroth；SH:Schroth & Hippotherapy Exercises; LY:Lyon; SB:Schroth & Balance Training; DS:Daoyin Spinal Balance Exercises; SS:Schroth & Sling; SSE:Spinal Strengthening Exercises; SWBV:Side-alternating Whole Body Vibration; PE:Pilates Exercise; AS:Active Self-correction and Task-oriented Exercises; SR:Schroth & Rehabilitation Exercises; SSIT:Schroth & Sensory Integration Training; SEAS:Scientific Exercise Approach to Scoliosis; BSPT:Barcelona Scoliosis Physical Therapy School; Con:Standard of Care.


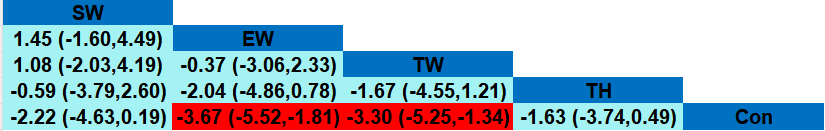


FIGURE 10. Pairwise comparison results of intervention period. SW:6-10 Weeks; EW：12-18 Weeks; TW：24-26 Weeks; TH：52 Weeks; Con:Intervention group.


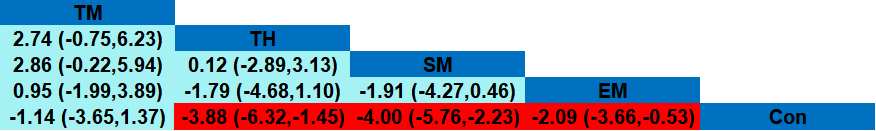


FIGURE 11. Pairwise comparison results of session duration. TM: 12-20 Min；TH：30-45 Min；SM：60 Min ；EM：80-90 Min ；Con:Intervention group.


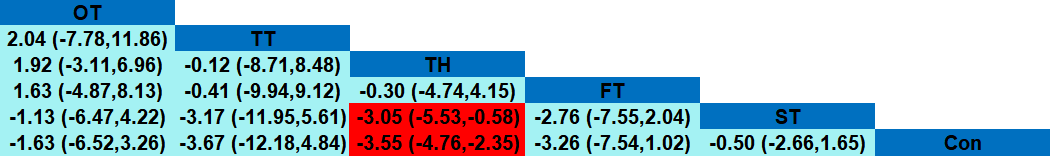


FIGURE 12. Pairwise comparison results of exercise frequency. OT：once per week; TT：twice per week; TH：three times per week; FT：five times per week; ST：seven times per week; Con:Intervention group.

**
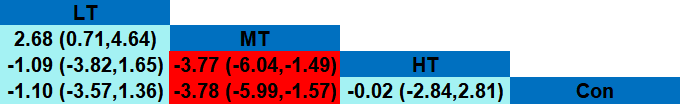
**

FIGURE 13. Pairwise comparison results of exercise intensity. LT:low intensity；MT：moderate intensity；HT： moderate-to-high intensity；Con:Intervention group.


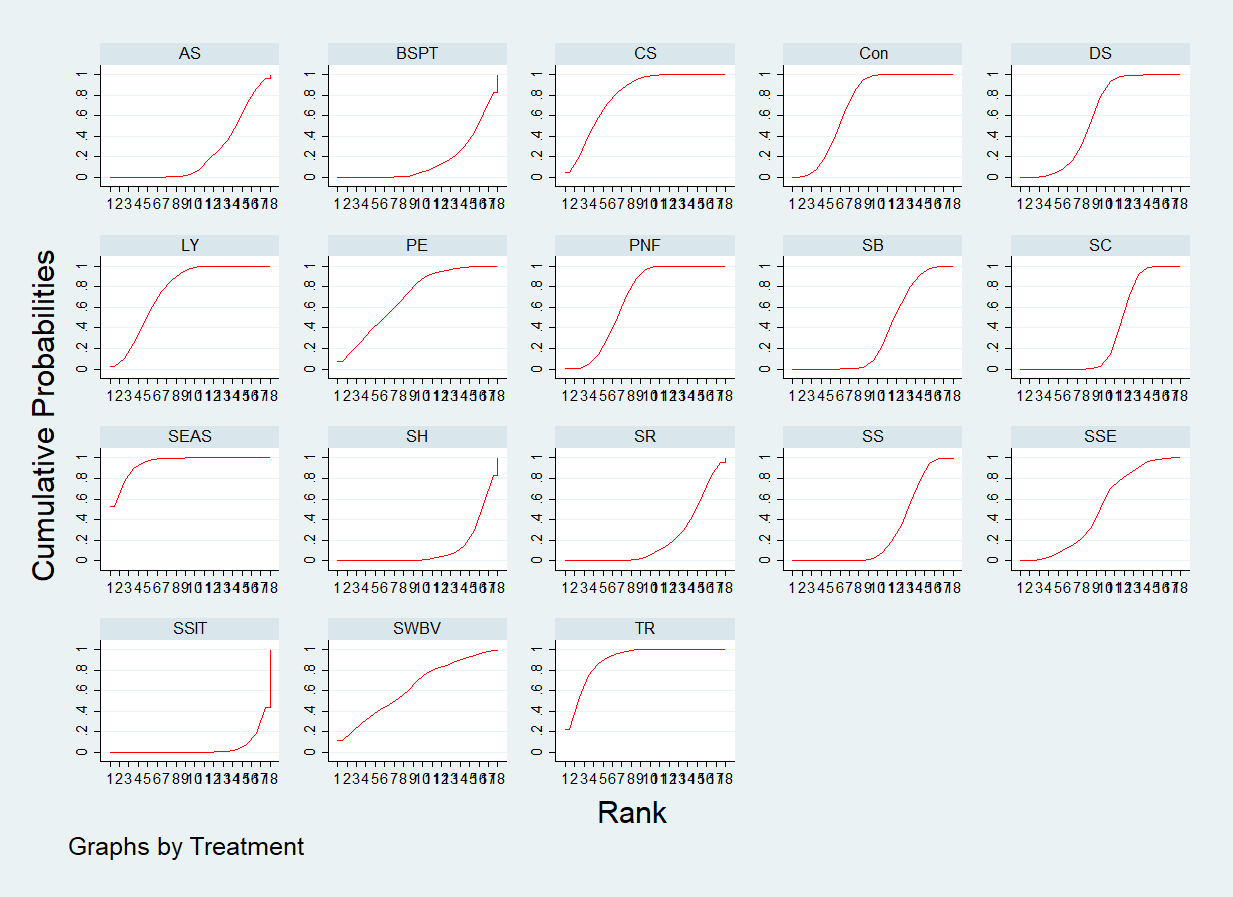


FIGURE 14. Ranking of cumulative probabilities for the optimal intervention by exercise type . CS:Core Stabilization Exercises; TR:Traditional Rehabilitation Exercises; PNF:Proprioceptive Neuromuscular Facilitation; SC：Schroth；SH:Schroth & Hippotherapy Exercises; LY:Lyon; SB:Schroth & Balance Training; DS:Daoyin Spinal Balance Exercises; SS:Schroth & Sling; SSE:Spinal Strengthening Exercises; SWBV:Side-alternating Whole Body Vibration; PE:Pilates Exercise; AS:Active Self-correction and Task-oriented Exercises; SR:Schroth & Rehabilitation Exercises; SSIT:Schroth & Sensory Integration Training; SEAS:Scientific Exercise Approach to Scoliosis; BSPT:Barcelona Scoliosis Physical Therapy School; Con:Standard of Care.


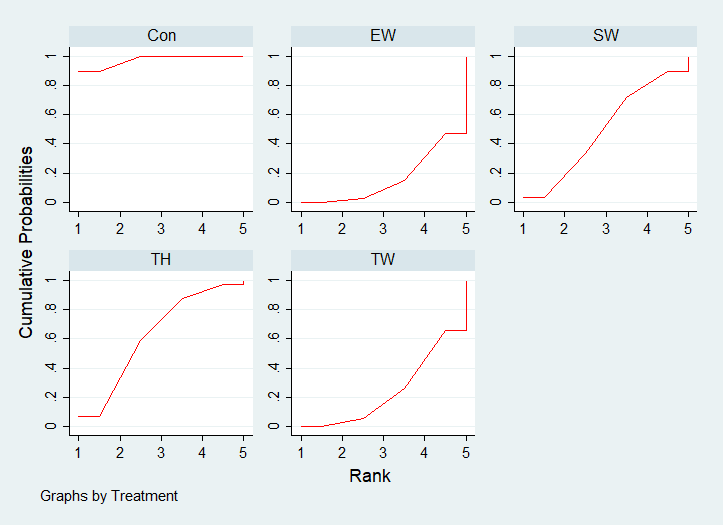


FIGURE 15. Ranking of cumulative probabilities for the optimal intervention by intervention period. SW:6-10 Weeks; EW：12-18 Weeks; TW：24-26 Weeks; TH：52 Weeks; Con:Intervention group.


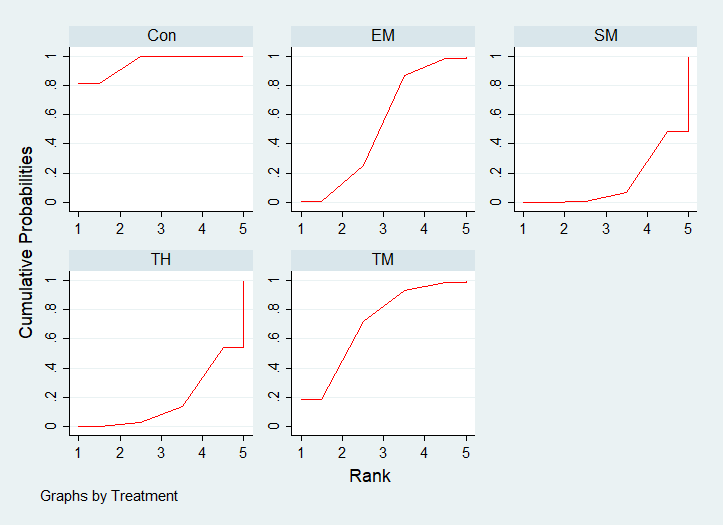


FIGURE 16. Ranking of cumulative probabilities for the optimal intervention by session duration.TM: 12-20 Min；TH：30-45 Min；SM：60 Min ；EM：80-90 Min ；Con:Intervention group.

**
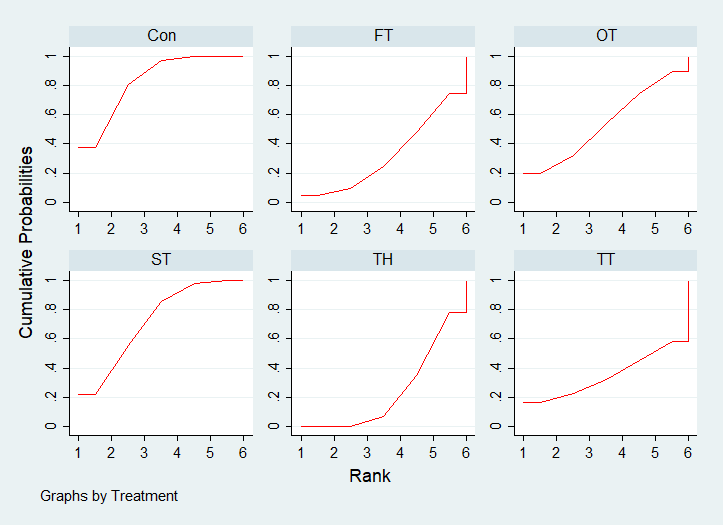
**

FIGURE 17. Ranking of cumulative probabilities for the optimal intervention by exercise frequency. OT：once per week; TT：twice per week; TH：three times per week; FT：five times per week; ST：seven times per week; Con:Intervention group.

**
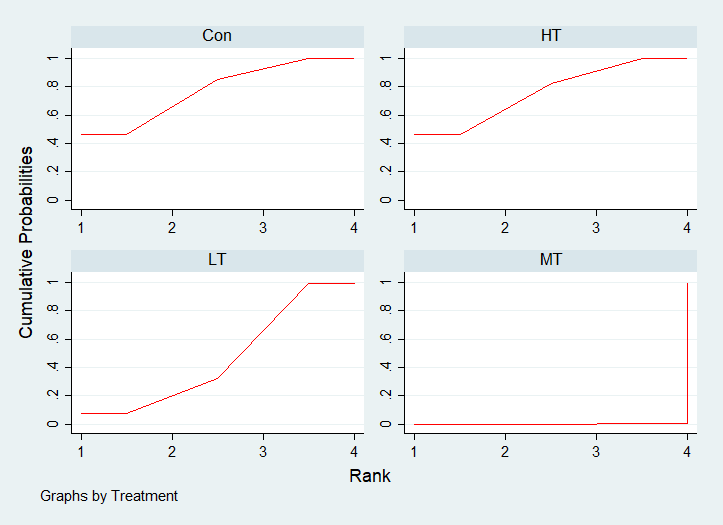
**

FIGURE 18. Ranking of cumulative probabilities for the optimal intervention by exercise intensity. LT:low intensity；MT：moderate intensity；HT： moderate-to-high intensity；Con:Intervention group.


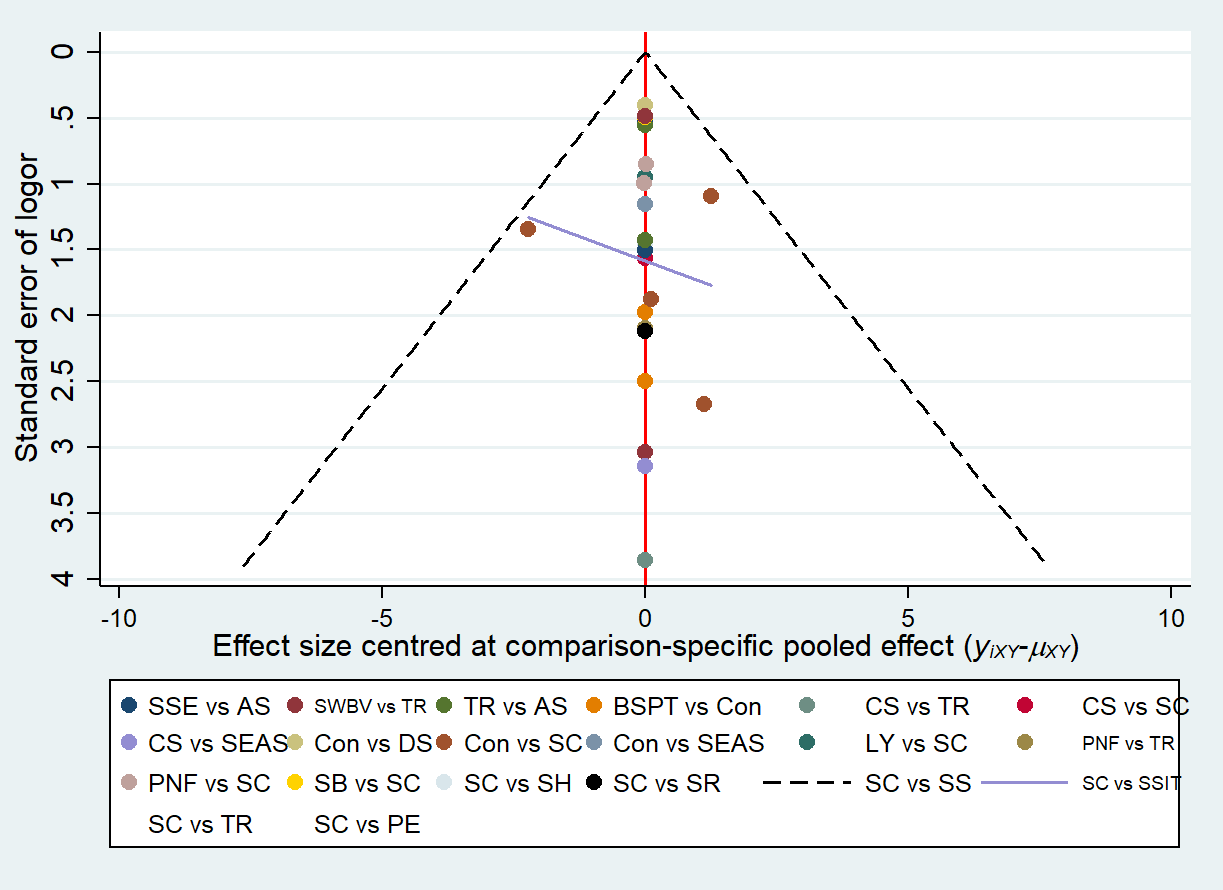


FIGURE 19. Comparison-adjusted funnel plot. CS:Core Stabilization Exercises; TR:Traditional Rehabilitation Exercises; PNF:Proprioceptive Neuromuscular Facilitation; SC：Schroth；SH:Schroth & Hippotherapy Exercises; LY:Lyon; SB:Schroth & Balance Training; DS:Daoyin Spinal Balance Exercises; SS:Schroth & Sling; SSE:Spinal Strengthening Exercises; SWBV:Side-alternating Whole Body Vibration; PE:Pilates Exercise; AS:Active Self-correction and Task-oriented Exercises; SR:Schroth & Rehabilitation Exercises; SSIT:Schroth & Sensory Integration Training; SEAS:Scientific Exercise Approach to Scoliosis; BSPT:Barcelona Scoliosis Physical Therapy School; Con:Standard of Care.
